# The Efficacy of Mindfulness-Based Cognitive Therapy as a Public Mental Health Intervention for Adults with Mild to Moderate Depressive Symptomatology: A Randomized Controlled Trial

**DOI:** 10.1371/journal.pone.0109789

**Published:** 2014-10-15

**Authors:** Wendy T. M. Pots, Peter A. M. Meulenbeek, Martine M. Veehof, Jorinde Klungers, Ernst T. Bohlmeijer

**Affiliations:** 1 University of Twente, Department of Psychology, Health & Technology, Enschede, the Netherlands; 2 Dimence, Community Mental Health Center, Almelo, the Netherlands; 3 GGNET, Community Mental Health Center, Warnsveld, the Netherlands; UNC Chapel Hill, United States of America

## Abstract

**Objective:**

Although there has been growing evidence for the efficacy of mindfulness-based cognitive therapy (MBCT) for different clinical populations, its effectiveness as a public mental health intervention has not been studied. The present study evaluates a community-based MBCT intervention for adults with mild to moderate depressive symptomatology in a large multi-site, pragmatic randomized controlled trial.

**Method:**

The participants with mild to moderate depressive symptomatology were recruited from the general population and randomized to the MBCT intervention (n = 76) or to a waiting list control group (n = 75). Participants completed measures before and after the intervention. Participants in the experimental condition also completed these measures at a 3-month follow-up.

**Results:**

In the experimental condition significant reductions in depression, anxiety, and experiential avoidance, and improvements in mindfulness and emotional- and psychological mental health were found, compared to the waiting list (effect sizes Cohen's d = 0.31–0.56). These effects were sustained at the 3-month follow-up. The likelihood of a clinically significant change in depressive symptoms was significantly higher for the MBCT group [odds ratio (OR) 3.026, p<0.01 at post-treatment; NNT = 5.10].

**Discussion:**

MBCT as a public mental health intervention for adults with mild to moderate depressive symptoms seems effective and applicable in a natural setting.

**Trial Registration:**

Nederlands Trial Register NTR2096

## Introduction

Minor depression is a highly prevalent disorder with a large negative impact on quality of life and yielding high economic costs [1]. Offering interventions for people with depressive symptomatology is a necessary public mental health strategy complementary to the treatment of depression in inpatient and outpatient settings [2]. One such effective strategy is to pro-actively offer treatments for people with mild to moderate symptoms of depression [3], [4]. However, a public mental health intervention needs to be attractive for people who don't suffer from severe symptoms of depression yet [5]. Using a positive framework and promoting positive mental health as well as reducing psychological distress offers opportunities to attract more people [6]–[8]. Mindfulness-based cognitive therapy (MBCT) may be such an intervention, as it focuses on promoting positive mental health instead of only focusing on the reduction of psychological distress. MBCT is an 8-week group-based training that combines meditation exercises with cognitive techniques. It was originally designed for prevention of relapse in people with recurrent depression [9]. In patients with three or more previous episodes of depression, MBCT significantly reduced the risk of relapse compared to the treatment as usual [10]–[12]. Moreover, in a recently published randomized controlled trial Williams et al. [13] found evidence that the number of episodes is a marker for those with greater vulnerability due to a history of childhood trauma and adversity. Strauss et al. [14] conducted a meta-analysis on mindfulness based interventions for people diagnosed with a current episode of an anxiety or depressive disorder and found that MBCT can also be effective for people who are currently depressed. MBCT targets processes such as avoidance of negative emotions and engaging with maladaptive thinking and rumination, that maintain depressive symptomatology in general [15], [16]. Mindfulness is often referred to as intentionally paying attention to present moment experiences in a non-judgemental way [17]. Awareness and acceptance of negative experiences will reduce experiential avoidance (EA) [15]. EA has been defined as the unwillingness to remain in contact with experiences such as feelings, thoughts, and bodily sensations, as an attempted means of behavioral regulation [15]. Psychological flexibility is the counterpart of experiential avoidance. As EA could be seen as an important factor that maintains depressive symptomatology, MBCT may also be effective as a public mental health intervention for people with depressive symptomatology. This fits with a growing interest in adapting MBCT to other psychiatric disorders, such as anxiety disorders, and bipolar disorder, but also treatment-resistant major depressive disorder (for a recent review see Chiesa & Serretti [18]). Research into the potential mechanisms of action in MBCT is in its infancy. In a recent review Chiesa and Serretti [19] suggested that mindfulness-based interventions may enhance positive emotion regulation strategies, as well as self-compassion levels, and decrease rumination and experiential avoidance. They suggested that these changes are associated with several clinical benefits, including the reduction of stress and depression levels, as well as the enhancement of positive emotions. Recently, Kaviani et al. [20] found that MBCT can be effective in a non-clinical population of female students in Iran. Also, Cavanagh et al. [21] adapted MBCT as a brief online intervention and found it to be effective in a non-clinical population of students. To our knowledge, there is no research on the effectiveness of a community-based MBCT intervention for adults with depressive symptomatology. In order to modify the original MBCT as a public mental health intervention a few changes were made in time-investment. We expected a community-based MBCT with a reduced weekly time-investment to be more acceptable for the target group.

The aim of this study was to evaluate the effect of mindfulness-based cognitive therapy modified as a public mental health intervention for depressive symptomatology, in a sample of self-referred adults with mild to moderate depressive symptomatology, and offered by community mental health centers. We evaluated the effect with respect to various psychological variables, such as depression, anxiety and positive mental health. We hypothesized that changes in depressive symptoms would be mediated by psychological flexibility and mindfulness. To strengthen the trial's external validity, the intervention was studied in its natural setting.

## Method

The protocol for this trial and supporting CONSORT checklist are available as supporting information; see Checklist S1 and Protocol S1. This study was approved by the METiGG, a medical ethics committee for research in mental health settings in the Netherlands. In addition, this study has been registered in the Nederlands Trial Register, the Primary Dutch register for clinical trials (NTR2096).

### Design

A pragmatic, multi-site, randomized controlled trial was conducted comparing MBCT with a control condition. Participants were randomly assigned after receiving their written consent, either to MBCT or to the waiting list control, by means of a centrally conducted randomization process executed by an independent researcher. The randomization was carried out for the two groups with stratification on gender, using a computer generated random sequence of numbers. The control condition consisted of a waiting list, where wait-listed people were free to use other kinds of care. The wait-listed participants knew that they could start the training after the experimental condition had completed the intervention, i.e. after 3 months. The study is pragmatic as it mimics the Dutch health care system as closely as possible in terms of patient recruitment, conducting intake, offering interventions, and monitoring outcomes.

### Participants and procedure

Participants were recruited from November 2009 until October 2010, through advertisements in regional newspapers, information booklets and general practitioners. Four Dutch community mental health centers, from both urban and rural areas, participated in the study. In the advertisements, distributed within the regions of the participating community mental health services, the target group was described as adults who were hindered by depressive symptoms. Applicants were referred to a specifically developed website, where they could find detailed information about the study. When interested, they were sent an information letter and an informed consent form. For screening, the standard procedures employed by the mental health institutions were used. The community mental health centers were responsible for the procedure, and the in- and exclusion criteria were examined by experienced mental health nurses on the basis of a checklist, under supervision of a clinical psychologist. The inclusion criteria were: adults of 18 years and over, presenting depressive symptoms. Applicants were excluded if diagnosed with a current severe major depressive episode (MDE; eight or nine out of a total of nine symptoms) or when having a moderate to high suicide risk, according to the Dutch version of the Mini International Neuropsychiatric Interview (MINI) [22], [23]. Other exclusion criteria were: receiving psychological or pharmacological treatment for mental complaints within the last three months, and presence of other severe mental or social problems warranting treatment or likely to interfere with participation in the group course. People meeting one of the exclusion criteria were advised to seek regular treatment.

### Power analysis

A sample of 60 participants per condition at post-intervention was needed to detect an effect size of 0.50 (Cohen's d) for the primary outcome with a statistical power of (1–β) = 0.80 in a two-tailed test (p<0.05). Taking into account a drop-out rate of 20%, 150 eligible participants were needed.

### Measures

Measurements were taken at baseline (T0), and at post-treatment after 3 months (T1). In order to study the stability of the effect of MBCT, the patients in the experimental group received a follow-up measurement (T2) at 6 months after baseline. For the control condition, the measurement at 6 months is a post-treatment measurement. All measurements had good psychometric properties and are frequently applied in international studies.

The primary outcome measure was depressive symptomatology, measured by the Dutch version of the Center of Epidemiological Studies - Depression Scale (CES-D; 20 items, score 0–60) [24]. Higher scores mean more depressive symptoms [24], [25].

Secondary outcome measures were anxiety symptoms and positive mental health. Anxiety was measured by the Hospital Anxiety and Depression Scale - Anxiety subscale (HADS-A; 7 items, score 0–21) [26] for assessing the presence and severity of anxiety symptoms. Higher scores mean more anxiety symptoms [26], [27]. Positive mental health was measured by the Mental Health Continuum - Short Form (MHC-SF) [28], that measures emotional well-being (3 items; score 0–15), social well-being (5 items; score 0–25) and psychological well-being (6 items; score 0–30). Higher scores indicate greater emotional, social, and psychological well-being [28], [29].

Measures of proposed processes of change included measures of EA and mindfulness. The Acceptance and Action Questionnaire-II (AAQ-II; 10 items, score 10–70) [30] was used to measure the willingness to be in contact with aversive internal experiences, to accept these events, and to pursue values in the presence of the experiences. Higher scores indicate lower levels of EA or higher levels of psychological flexibility [30], [31]. The Five Facet Mindfulness Questionnaire (FFMQ) [32] was used to measure mindfulness in five sub-dimensions: (1) observing (8 items), defined in terms of noticing or attending to internal and external experiences; (2) describing (8 items), defined in terms of labelling internal experiences with words; (3) acting with awareness (8 items), defined in terms of attending to one's activities of the moment (opposite of acting on automatic pilot); (4) non-judging of inner experience (8 items), defined in terms of taking a non-evaluative stance toward thoughts and feelings; and (5) and non-reactivity to inner experience (7 items), defined in terms of allowing thoughts and feelings to come and go, without getting caught up in or carried away by them. Facet scores range from 8 to 40 (except for the non-reactivity, which ranges from 7 to 35), with higher scores indicating more mindfulness [32], [33].

To evaluate the level of satisfaction of the participants after the intervention, a self-developed evaluation questionnaire was used, including a question on how the participants evaluated the program using a scale from 1(very poor) to 10 (excellent).

### Waiting list control

Participants in the control condition participated in the MBCT training after a 3-month waiting list period. Participants were instructed to seek help from their general practitioner, family or other sources, as they normally would, should they encounter symptomatic deterioration or other difficulties during the waiting list period.

### Intervention

In this study MBCT was delivered according to the guidelines of Segal et al. [9]. The original training was adapted to suit a public mental health approach. The intervention was aimed at people with mild to moderate symptoms of depression. In order to lower the threshold for people without severe distress to participate in MBCT, the participants were asked to practice meditations for 15 minutes a day instead of the original 45 minutes a day, and the sessions were limited to 1, 5 hours instead of the original 2, 5 hours. To ensure that all of the elements of the original course were preserved, the eight-session training was extended to 11 sessions. Key themes of the sessions included awareness (sessions 1, 2, 3), acceptance (sessions 5, 7, 9, 10) and disengaging from thoughts (sessions 4, 6, 8), with the last session (session 11) focusing on evaluation and integration. The training teaches skills to become more aware of, and to relate differently to thoughts, feelings and bodily sensations. A core feature of the training is to learn to become aware of, and disengage from habitual dysfunctional (cognitive) routines, to stop reacting automatically to internal experiences, and to act more ‘mindfully’. During the period of the training, the program consisted of daily homework exercises. The exercises were aimed at increasing attention to present moment experiences in a non-judgmental way, together with exercises designed to integrate application of awareness skills into daily life. To support homework assignments, participants received weekly homework registration forms, guided (taped) and unguided meditations, and information in a booklet. Group sizes varied between eight and 15 participants.

### Therapists

The MBCT instructors were all experienced psychologists and mental health nurses, with extensive former training in the original MBCT protocol by Segal [9] and Group psychotherapy. The trainers were also experienced meditators, with meditation experience ranging from 2 to 15 years.

### Statistical analyses

The statistical analyses were performed using SPSS 18. The data was analyzed on an intention-to-treat (ITT) basis. Missing values at baseline, post-intervention and follow-up were imputed with the use of SPSS Missing Value Analysis on the continuous measures with the expectation-maximization (EM) method. This method computes missing values based on maximum likelihood estimates using observed data in an iterative process [34]. The total percentage of missing data (T0-T1-T2) was 5%, due to unanswered items (0,6%) and incomplete assessments (4,4%). A comparison of results based on the imputed intention-to-treat sample versus the observed data revealed similar outcomes. Therefore, only the results from the intention-to-treat analyses are reported.

Independent sample t-tests and chi-square tests were conducted to examine differences between the two groups at baseline on sociodemographic variables and outcome measures. Analysis of covariance (ANCOVA) was conducted with depressive symptoms post-treatment as the dependent variable, treatment group as the independent variable, and five covariates consisting of pre-treatment depression score and four dummy-coded variables for each treatment site. The same analyses were conducted for the secondary measures. Analysis of variance (ANOVA) was conducted with process measures post-treatment as the dependent variable and treatment group as the independent variable. Assumptions for performing parametric analysis of (co)variance were all met. To investigate whether the effects in the intervention condition were maintained at follow-up paired-sample t-tests were carried out, comparing the scores on the follow-up (T2) with those at baseline (T0). Also, paired-sample t-tests were carried out for the control group, to measure the effect of the MBCT intervention after the waiting time period, comparing scores at post-treatment (T2 for the control group) with those at pre-treatment (T1 for the control group).

Effect sizes at post-treatment (T0-T1) were calculated with Cohen's d using the means and the pooled standard deviations of the measurements in the conditions. For the effect sizes at follow-up (T0-T2) the Cohen's d was corrected for dependence among means by using the correlation between the two means [35]. To interpret Cohen's d an effect size of less than 0.33 is considered small, while 0.33 to 0.55 is considered moderate and effect sizes of 0.56 to 1.2 are considered large [36]. Comparisons were two-tailed and interpreted with a significance value of p<0.05.

With the Jacobson and Truax methodology, the proportion of participants was determined who made a clinically significant change on the CES-D from baseline to post-treatment [37]. First, the reliable change was calculated with the reliable change index (RCI). Jacobson and Truax suggest that subjects can be considered to have improved when they shift from a dysfunctional distribution to a functional one, and the reliable change scores exceed measurement error (calculated by dividing the difference between the pretest and posttest scores by the standard error of the measurement). Second, the recovery criterion was defined as a post-treatment score below the cut-off value of 16 for clinically relevant depressive symptoms [38], [39]. Because we studied a population with a mild to moderate symptomatology, the mean score at baseline was already at the recovery criterion (M = 16.04; range 1–43; S.D. = 8.08). A clinically significant change on the CES-D is thus defined as having a reliable change between the measurements, which required a post-treatment score below the cut-off of 16. Participants that had a clinically significant change were either coded 1 (implying a favorable treatment response, ‘success’) or 0 (‘failure’). The binary outcome was used to calculate the odds ratio (OR) using logistic regression. Based on the clinically significant change proportions, the number needed to treat (NNT) was calculated [40]. To provide a more complete representation of the effects of the intervention, the outcomes were also analyzed for intervention completers only (somewhat arbitrarily defined as participants that attended at least 9 sessions).

The process measures were expected to be mediators between the MBCT intervention and post-treatment levels of depressive symptomatology (CES-D). Mediation was performed for all process measures that were significantly different between the intervention and control condition in the ANOVA. Then, all steps outlined by Baron and Kenny [41] were used. In the first step linear regression analysis was performed, with treatment group as independent variable and depressive symptoms post-treatment as the dependent variable. In the second step we tested the effect of the independent variable on the proposed mediators with linear regression analyses, with treatment group as independent variable and the residual change scores of the proposed mediators as the dependent variables. The third step tested the effect of the proposed mediator on the dependent variable. The indirect effect of the mediator on the outcome was assessed to examine whether an increase in psychological flexibility and mindfulness during the intervention would mediate the effects of the intervention on depressive symptomatology at post-intervention. Simple mediational analyses with bootstrapping procedures (n = 5000 bootstrap resamples) were used to assess the indirect effect of the mediator on the outcome [42]. An indirect effect was considered significant in the case zero was not contained in the 95% confidence interval.

## Results

### Enrollment, treatment adherence, satisfaction and drop-out

**Figure 1:**
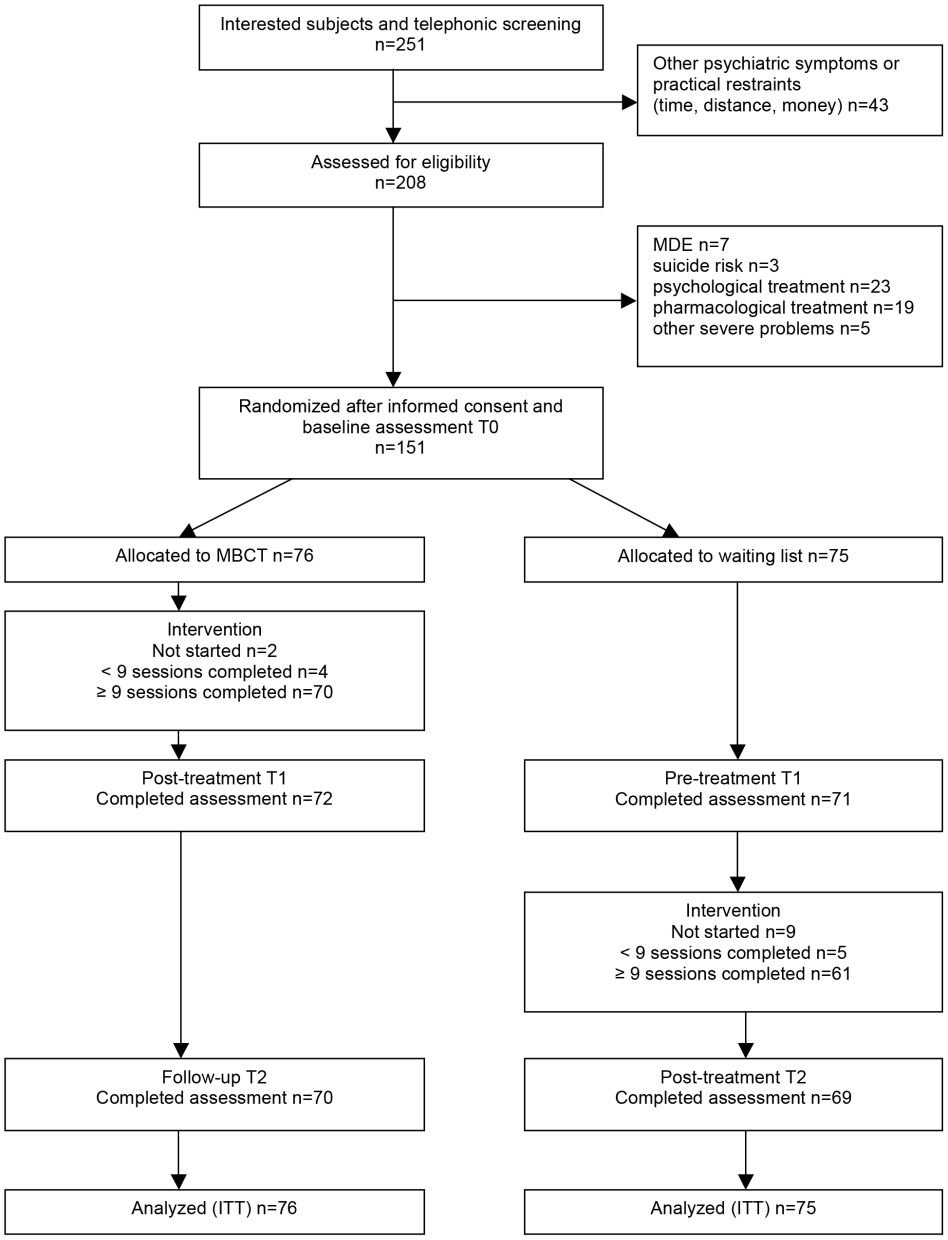
Participant flow. MDE  =  major depressive episode.

Figure 1 provides an overview of the flow of participants. A total of 251 persons were interested in the training. During telephone screening, 43 persons presented other psychiatric symptoms or practical restraints that precluded them from participation in the trial. The remaining 208 applicants were assessed for eligibility. Through interviewing, a further 57 were excluded. After signing the informed consent form, the included 151 participants were randomly assigned to the MBCT intervention (n = 76) and the waiting list condition (n = 75).

After randomization, two participants decided not to start with the intervention due to time constraints (n = 1) and health problems (n = 1). Four other participants in the MBCT (8%) group did not complete the intervention (attendance of at least 9 sessions). The reasons given for non-completion were that the intervention was too time consuming (n = 1), practicality reasons (n = 1), insufficient concentration (n = 1), and an unknown reason (n = 1). Two of the four community mental health centers evaluated the course resulting in anonymous evaluation forms of all participants, including those who did not participate in the study. Based on these results overall the intervention was evaluated as positive on a scale from 1 to 10 (m = 7.98, sd = 0.72, n = 130).

At T1 (post-treatment for the MBCT group and pre-treatment for the waiting list group), data was available for 143 participants (drop-out rate 5.3%) and at T2 (follow-up for the MBCT group and post-treatment for the waiting list group), data was available for 139 participants (drop-out rate 7.9%). There were no significant differences at baseline on all the measurements between participants who completed the assessments and those who did not complete all measures.

### Baseline characteristics

Table 1 shows an overview of the participants' characteristics. Participants had a mean age of 48 years (SD = 11.29, range 20–81) and were predominantly female (78.1%). The majority was of Dutch origin (96%), had a paid job (66.9%), and were living with a partner (75.5%). The level of education was high for 65.5% of the participants and intermediate for 30.5%. At the entry of the study, 2.6% met the criteria of mild MDE and 4% were diagnosed with moderate MDE. There were no significant differences at baseline between the MBCT group and the control group for any of the demographic variables or outcome measurements. Also, there were no significant changes in the waiting time period for the control group, comparing scores at baseline (T0) with pre-treatment measurement (T1), on any of the outcome measures.

**Table 1:** Baseline characteristics of the total sample, MBCT intervention and waiting list control condition.

		MBCT (n = 76)		Waiting list (n = 75)		Total (n = 151)	
		n	%	n	%	n	%
*Age* a	Mean (SD)	47.99 (10.59)		47.88 (12.02)		47.93 (11.29)	
	Range	24–74		20–81		20–81	
*Gender* b	Female	59	77,6	59	78,7	118	78,1
	Male	17	22,4	16	21,3	33	21,9
*Marital status* b	Single	17	22,4	20	26,7	37	24,5
	**Living with partner**	59	77,6	55	73,3	114	75,5
*Race* b	Dutch	71	93,4	74	98,7	145	96,0
	Other	5	6,6	1	1,3	6	4,0
*Education* b	Low	2	2,6	4	5,3	6	4,0
	Middle	21	27,6	25	33,3	46	30,5
	High	53	69,7	46	61,3	99	65,5
*Employment* b	Paid	53	69,7	48	64,0	101	66,9
	No paid	23	30,3	27	36,0	50	33,1
*Diagnosis* b	Mild MDE	3	3,9	1	1,3	4	2,6
	Moderate MDE	0	0,0	6	8,0	6	4,0

*Note*. MDE, major depressive episode.

a No significant differences between intervention and control condition (t-test with *P*<0.05).

b No significant differences between intervention and control condition (chi-square-test with *P*<0.05).

## Treatment effects

### Primary outcome

The means and standard deviations for the primary outcome measure, the results of the ANCOVA and the effect sizes are presented in Table 2. Compared to the control condition, participants in the intervention condition reported significantly decreased depressive symptoms at post-treatment (Table 2). The effects of the intervention condition on depressive symptoms were maintained at follow-up compared to baseline measurement [t(75) = −3.46, *p*<0.01]. Moderate effect sizes were found at post-treatment (*d* = 0.50) and follow-up (*d* = 0.40). The control group also showed significant reductions in depression after they received the intervention at T2 [t(74) = −3.03, *p*<0.01].

**Table 2:** Means and standard deviations for outcome measures and results of ANCOVA for intervention effects, and Cohen's d.

Measures	MBCT		Waiting list				
	(n = 76)		(n = 75)				
	M^a^	SD^a^	M^a^	SD^a^	F(df)^b^	*P*	*d*
*Primary outcome*							
CES-D baseline	15.62	7.86	16.46	8.32			
CES-D post-treatment	11.79	8.76	16.43	9.94	10.91(1)	0.001	0.50
CES-D follow-up^c^	11.77	8.25	13.60	8.88			
*Secondary outcomes*							
HADS-A baseline	7.96	3.40	8.57	3.58			
HADS-A post-treatment	6.14	3.52	8.22	3.89	12.42(1)	0.001	0.56
HADS-A follow-up^c^	4.88	2.88	6.54	3.84			
MHC-SF-EM baseline	3.15	0.95	3.15	0.78			
MHC-SF-EM post-treatment	3.46	0.85	3.19	0.88	5.32(1)	0.023	0.31
MHC-SF-EM follow-up^c^	3.54	0.95	3.61	0.78			
MHC-SF-SOC baseline	2.38	1.04	2.28	0.96			
MHC-SF-SOC post-treatment	2.72	1.08	2.49	1.05	1.62(1)	0.206	0.22
MHC-SF-SOC follow-up^c^	2.91	1.12	2.85	0.86			
MHC-SF-PSY baseline	2.92	0.89	2.96	1.00			
MHC-SF-PSY post-treatment	3.26	0.98	2.90	1.11	8.60(1)	0.004	0.34
MHC-SF-PSY follow-up^c^	3.37	0.93	3.41	0.89			

*Note*. ANCOVA, Analysis of covariance; CES-D, Center for Epidemiologic Studies - Depression.

Scale; HADS-A, Hospital Anxiety and Depression Scale - Anxiety subscale; MHC-SF, Mental Health Continuum - Short form; EM, emotional; SOC, social; PSY, psychological.

a Unadjusted condition means and standard deviations (SD).

b F-value, corrected for baseline values.

c Follow-up for intervention group and post-treatment for waiting list group.

### Clinically significant change

The reliable change on the CES-D appeared to be a pre-post difference of at least 7 scale points. Clinically significant change was thus defined as a recovery condition of a score ≤16 points on the CES-D (n = 138) and a RCI of 7 points. The proportion of participants with a score of ≥7 at T0 that reached a clinically significant change was 24/70 (34%) in the intervention group, versus 10/68 (15%) in the control condition [OR 3.026, 95% confidence interval (CI) 1.316–6.961, p<0.01, NNT = 5.10, under an intention-to-treat analysis]. These results compare well with completers-only findings: OR 2.916, 95% CI 1.252–6.795, p<0.01, NNT = 5.26.

### Secondary outcome measures

The means and standard deviations for the secondary outcome measures, the results of the ANCOVA and the effect sizes are presented in Table 2. Compared to the control condition, participants in the intervention condition reported significantly decreased anxiety symptoms after the intervention. At follow-up, the effects of the intervention condition on anxiety symptoms were maintained compared to baseline measurement [t(75) = −8.40, *p*<0.001]. The effect sizes for anxiety symptoms at post-treatment (*d* = 0.56) and at follow-up (*d* = 0.97) were large. The control group also showed significant reductions in anxiety after they received the intervention at T2 [t(74) = −5.15, p<0.001].

At post treatment, significant improvements in emotional well-being and psychological well-being were found. The effects of the intervention condition on emotional and psychological well-being were maintained at follow-up compared to baseline measurement [emotional well-being t(75) = 4.13, p<0.001; psychological well-being t(75) = 5.20, p<0.001]. Effect sizes at post-treatment were small (emotional well-being, *d* = 0.31) to moderate (psychological well-being, *d* = 0.34). The effect sizes at follow-up were moderate (emotional well-being, *d* = 0.50) to large (psychological well-being, *d* = 0.56). No significant effects were found at post-treatment for social well-being, with a small effect size (*d* = 0.22). At follow-up, there was a significant increase in social well-being compared to baseline measurement [t(75) = 5.58, p<0.001], with a large effect size (*d* = 0.63).

The control group showed significant reductions on positive mental health after they received the intervention at T2 [social well-being t(74) = 4.79, p<0.001; emotional well-being t(74) = 5.47, p<0.001; psychological well-being t(74) = 6.21, p<0.001].

### Process measures

Table 3 shows all process measurements, the results of the ANOVA, and the effect-sizes. Compared to the control condition, participants in the intervention condition showed significant improvement in psychological flexibility and all mindfulness facets (except for FFMQ-Describe). The effects of the intervention condition on all process measures were maintained at follow-up compared to baseline measurement [psychological flexibility, t(75) = 6.17, p<0.001; FFMQ observing, t(75) = 7.67, p<0.001; FFMQ describing, t(75) = 6.82, p<0.001; FFMQ acting with awareness, t(75) = 6.20, p<0.001; FFMQ non-judging of inner experience, t(75) = 6.46, p<0.001; FFMQ non-reactivity to inner experience, t(75) = 8.97, p<0.001]. The effect sizes post-treatment were small to large (*d* = 0.13–0.84) and large at follow-up (*d* = 0.71–1.03).

**Table 3:** Means and standard deviations for process measures, results of ANOVA and Cohen's d for intervention effects.

Measures	MBCT		Waiting list				
	(n = 76)		(n = 75)				
Process outcomes	M	SD	M	SD	F(df)	*P*	*d*
AAQ-II baseline	44.28	8.42	43.03	7.86			
AAQ-II post-treatment	49.59	8.80	44.36	10.01	11.62(1)	0.001	0.56
AAQ-II follow-up^a^	50.93	9.04	48.91	8.89			
FFMQ - Observe baseline	24.77	5.58	25.21	5.77			
FFMQ - Observe post-treatment	27.98	5.15	25.28	6.29	8.34(1)	0.004	0.47
FFMQ - Observe follow-up^a^	28.46	4.54	29.07	4.74			
FFMQ - Describe baseline	26.39	6.14	27.43	6.89			
FFMQ - Describe post-treatment	28.60	5.56	27.80	6.35	0.68(1)	0.410	0.13
FFMQ - Describe follow-up^a^	29.24	6.03	29.77	6.76			
FFMQ - ActAware baseline	20.92	5.45	22.61	5.48			
FFMQ - ActAware post-treatment	25.31	5.53	22.67	6.05	7.86(1)	0.006	0.46
FFMQ - ActAware follow-up^a^	25.08	5.51	26.31	5.30			
FFMQ - NonJudge baseline	23.64	6.04	22.87	5.48			
FFMQ - NonJudge post-treatment	27.68	5.16	24.72	5.66	11.28(1)	0.001	0.55
FFMQ - NonJudge follow-up^a^	28.16	5.57	28.05	5.52			
FFMQ - NonReact baseline	19.73	4.29	19.31	4.29			
FFMQ - NonReact post-treatment	23.86	4.27	20.24	4.32	26.82(1)	0.000	0.84
FFMQ - NonReact follow-up^a^	24.24	4.04	23.54	4.22			

*Note*. AAQ-II, Acceptance and Action Questionnaire-II; ANOVA, Analysis of variance; FFMQ, Five Facet Mindfulness Questionnaire; Observe, observing; Describe, describing; ActAware, acting with awareness; NonJudge, non-judging of inner experience; NonReact, non-reactivity to inner experience.

a Follow-up for intervention group and post-treatment for waiting list group.

### Mediational analyses

**Figure 2:**
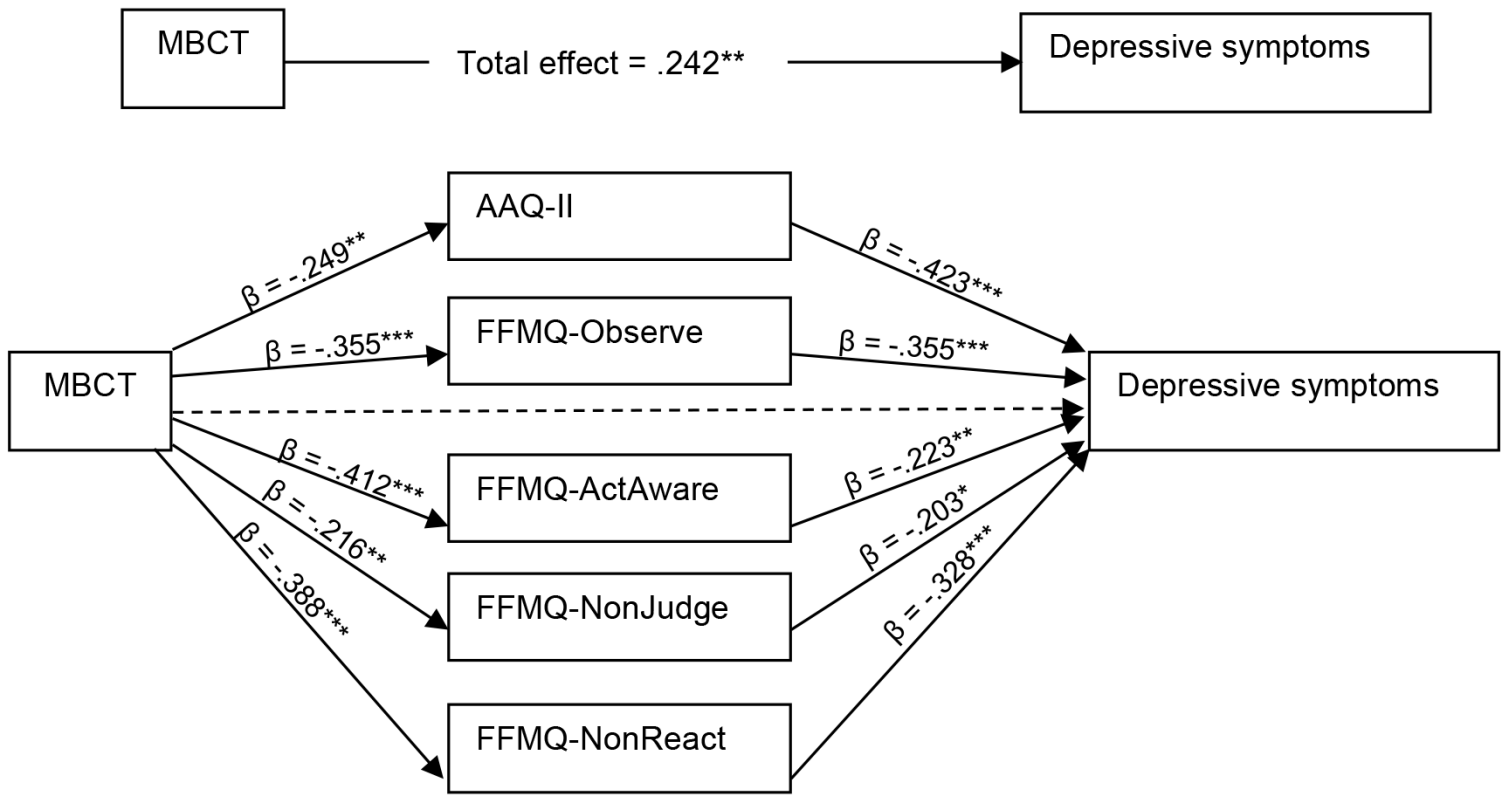
Mediation model of psychological flexibility and mindfulness as mediators. *Note*. AAQ-II, Acceptance and Action Questionnaire-II; FFMQ, Five Facet Mindfulness Questionnaire; Observe, observing; ActAware, acting with awareness; NonJudge, non-judging of inner experience; NonReact, non-reactivity to inner experience. ^*^ *P*<0.05; ^**^ *P*<0.01; ^***^ *P*<0.001.

Figure 2 shows the results of the first three steps of mediation. The FFMQ-Describe was excluded from the mediational analyses, having no significantly different effect in the ANOVA. The first step shows that the intervention condition had significantly decreased depressive symptoms at post-treatment, compared to the control condition. Step two shows that the intervention condition was significantly improved in psychological flexibility and mindfulness compared to the control condition. In step 3, all change scores of the process measures were significantly associated with the scores on the CES-D at post-treatment. In the last step of the mediational analysis, following Preacher & Hayes [42], results showed that the effect of the intervention on depressive symptoms was mediated by all process measures. Full mediation was found for improvement of psychological flexibility (direct effect β = .145, p = 0.057; indirect effect β = −.387, 95% CI 0.89–3.45), and mindfulness facets Observing (direct effect β = 0.132, p = 0.106; indirect effect β = −.308, 95% CI 0.98–3.60), and Non-reactivity to inner experience (direct effect β = .135, p = 0.108; indirect effect β = −.275, 95% CI 0.83−3.73). Partial mediation was found for improvement of mindfulness facets Acting with awareness (direct effect β = .176, p = 0.044; indirect effect β = −.160, 95% CI 0.18–2.84), and Non-judging of inner experience (direct effect β = .208, p = .011; indirect effect β = −.158, 95% CI 0.06–1.74).

## Discussion

### Main findings

To our best knowledge, this is the first study that evaluated MBCT as a public mental health intervention for adults with mild to moderate depressive symptomatology within a large pragmatic multi-site trial. The potential of offering MBCT to the community as a public mental health intervention is of importance, as the idea that MBCT is only effective in patients suffering from recurrent depression is superseded. In the present study the participants suffered from mild to moderate depressive symptoms but differed from severe clinical samples, as indicated by the substantially lower mean degree of severity of depressive symptoms as measured by the CES-D and the MINI. This corresponds to the nature and structure of the community-based MBCT, offered in our study. The results from this study indicate that MBCT as a public mental health intervention is effective in reducing depressive symptoms. We found a moderate effect size for depressive symptoms at post-treatment in comparison with the control group (d = 0.50). This effect size is comparable to the effect size of 0.42 that was found for psychological treatments on subthreshold depression [43]. The only studies of MBCT in community-based samples to our knowledge are from Kaviani et al. [20] and Cavanagh et al. [21]. Both conducted a randomized controlled trial in a sample of students comparing MBCT to a waiting list control group. They showed significant reductions of depressive and anxiety symptoms over time, with effect limited by low generalizability. Our study corroborates with these findings, further showing that MBCT seems to be effective in a population of adults with mild to moderate depressive symptomatology, and that MBCT can be used as a public mental health intervention in the community. Our finding that the results are maintained at 3 months follow-up is promising, but needs to be substantiated by longer follow-up measurements under controlled conditions. Offering a public mental health intervention in a positive framework might be less stigmatizing for participants with depressive symptomatology or minor depression [5]. MBCT focuses on the enhancement of promoting positive skills and therefore has the potential to offer an alternative to stigmatization.

The likelihood of a clinically significant change in depressive symptomatology in our study was substantially higher in the intervention condition compared to the waiting-list control group. As the presence of clinically relevant depressive symptoms is known to be an important risk factor for clinical depression [44], this outcome suggests that MBCT implemented as a public mental health intervention for adults with mild to moderate depressive symptomatology may decrease the risk of developing a MDE. It is shown that the effects on clinical cases of interventions for people with sub-clinical symptomatology are most prominently found after longer periods of time [45]. However this result needs to be corroborated with longer follow-up measurement and the use of diagnostic instruments as outcome measures.

Moreover, the MBCT intervention resulted in significant reductions in anxiety symptoms with large effect sizes post-treatment and at the 3-month follow-up. The effect of MBCT on anxiety symptoms are consistent with the meta-analysis by Vøllestad et al. [46], which found a large effect size (g = 0.83) for controlled studies of mindfulness and acceptance-based interventions for patients with anxiety symptoms. Strauss et al. [14] found no effects on anxiety symptom severity in their meta-analysis, applying a more stringent definition and excluding trials with interventions based on Acceptance and Commitment Therapy. Our study supports the findings of Vøllestad et al. [46], indicating that MBCT as a public mental health intervention could be effective in not only reducing depressive symptoms, but also in significantly reducing anxiety symptoms post-treatment and, even more substantially, at the 3-month follow-up. As anxiety symptoms often coexist with depression and may precipitate depression [47], these findings could indicate that the application of MBCT has the potential to further decrease the incidence of depression. However, this needs to be substantiated by further research.

The results also show a significant effect on positive mental health. It thus seems that MBCT has the potential not only to reduce psychological distress, but also to improve emotional, psychological, and social (only at follow-up) well-being as well. This finding confirms earlier studies that show that MBCT can promote well-being in patients with anxiety or depression [20], [48]. The effects of MBCT on positive mental health are of importance. There is growing evidence that positive mental health and psychopathology are related but different dimensions of mental health [49], and that positive mental health is a protective factor against mental illnesses [6], [7]. Several researchers suggest that aspects of psychological well-being (e.g. meaning, mastery, autonomy, goals) increase personal resilience [50]–[52]. The findings of this study suggest that adults with mild to moderate depressive symptomatology that participated in this public mental health intervention will be able to better cope with life adversity in the future. Moreover, offering a public mental health intervention in a positive framework might be less stigmatizing for participants with depressive symptomatology [5].

Mediational analyses show that the efficacy of MBCT compared to the control group on reducing post-treatment depressive symptoms is mediated by an increase in psychological flexibility and all mindfulness skills, except for the subscale ‘describe’. Our results are in line with Kuyken et al. [53], which showed that 15-month follow-up level of depression were mediated by mindfulness skills and self-compassion. Developing a compassionate attitude toward one's own negative thoughts and feelings mediated the effect of MBCT on depressive symptoms and relapse. These findings are also in line with earlier studies that demonstrated the association between low psychological flexibility (i.e. experiential avoidance) and mindfulness and psychopathology [15], [54], [55]. The increase of psychological flexibility and mindfulness that mediated the effects of MBCT on depressive symptomatology suggests that participants have gained additional adaptive emotion regulation skills in response to negative affect-producing stressors [19], [54].

The original MBCT training was designed for people with a history of depression and requires participants to commit to a 2.5-hour group session and to 45 to 60 minutes of mindfulness practice each day for 8 weeks [9]. The current study suggests that mindfulness exercises of a total of 15 minutes a day may be effective for adults with mild to moderate depressive symptomatology. Much of the research on the effects of MBCT is conducted based on a clinical population. For the non-clinical population, mostly still working and active socially, the required time commitment on a weekly and daily basis may be a barrier to effectively integrate exercises into daily life. Carmody and Baer [56] concluded in their review that the correlation between mean effect size and number of in-class hours was non-significant for both clinical and non-clinical samples. They suggested that adaptations that include less class time may be worthwhile for populations for whom reduction of psychological distress is an important goal and for whom longer time commitment may be a barrier to their ability or willingness to participate. Our findings are in line with a number of studies which have shown that short-term meditation can lead to more tolerance, and a lower distress of pain and perceived stress (e.g. [57], [58]). For example, Klatt et al. [57] showed that a 60-minute training together with 20 minutes of daily practice of meditation can have a significant positive effect on levels of perceived stress in healthy working adults (*p* = .0025). Also, Cavanagh et al. [21] effectively adapted their online MBCT to a brief intervention with daily mindfulness meditation practices of 10 minutes. Our finding that an 11-week MBCT training with 15 minutes of daily mindfulness meditation practice can sufficiently and significantly reduce depressive symptomatology seems promising from a public mental health perspective and is in line with Carmody and Baer [56]. The finding that the community-based MBCT intervention was very positively evaluated by the participants (scoring 7.98 out of 10), and that very few people dropped out of the intervention or dropped out of the study, underscores the feasibility and the attractiveness of the intervention.

### Limitations

Some limitations must also be acknowledged. First, for the design of the study as a waiting-list compared RCT, controlling for the influence of possible non-specific factors, such as attention and social interaction, was unlikely. Future research should use an active control intervention or an attention placebo controlled design to overcome this limitation. Secondly, the study used a short follow-up period of 3 months for which the follow-up was limited to a within group analysis. For ethical reasons, the time until the control group could receive the intervention was limited to 3 months. For future research, a longer follow-up (e.g. 1-year follow-up) is recommended to study the impact of MBCT on the incidence of depression. Third, the design was a pragmatic randomized controlled trial with self-referred participants, so the results of the study may have been influenced by a selection bias. All measures were self-report: no psychiatric diagnoses were available because participants were recruited from the general public. Generalizability of the findings to patients seeking treatment cannot be assumed. On the other hand, no restrictions were made to the level of depressive symptoms, as is customary to the procedures applied in community mental health centers. In this regard, the study was representative for standard general practice.

## Conclusion

This study shows that MBCT as a public mental health intervention for adults with mild to moderate depressive symptomatology is effective by not only reducing depressive symptoms and anxiety symptoms, but also enhancing positive mental health and psychological flexibility. Furthermore, this study shows that the intervention is applicable and effective in a natural setting.

## Supporting Information

Checklist S1 CONSORT Checklist.
(PDF)

File S1 Metadata file.
(PDF)

Protocol S1 Trial protocol English translation.
(PDF)

Protocol S2 Trial protocol original Dutch language.
(PDF)
