# Supplementary material for: The Efficacy of Mindfulness-Based Cognitive Therapy as a Public Mental Health Intervention for Adults with Mild to Moderate Depressive Symptomatology: A Randomized Controlled Trial
Source: PLoS One. 2014 Oct 15;9(10):e109789. doi: 10.1371/journal.pone.0109789 (PMC4198116; doi:10.1371/journal.pone.0109789)
Supplement: File S1 — Metadata file. (PDF) [file pone.0109789.s002.pdf]

Details dataset

Title Dataset

Dataset Pots et al_MBCT as a public mental health intervention_PlosOne_24072014

Creators

W.T.M. Pots, P.A.M. Meulenbeek, M.M. Veehof, J. Klungers, E.T. Bohlmeijer

Contact information

W.T.M. Pots
University of Twente
Department of Psychology, Health & Technology
Drienerlolaan 5, PO Box 217, 7500 AE,
Enschede, The Netherlands.

Persistent Identifier

urn:nbn:nl:ui:13-6gd4-iv

Data Citation

When using this data, please cite the dataset and the original article.

Citation dataset: Pots, W.T.M. (2011). The efficacy of mindfulness-based cognitive therapy as a public mental health intervention for adults with mild to moderate depressive symptomatology: a Randomized Controlled Trial. [Data file and Metadata file]. Online archiving system DANS-EASY. Persistent Identifier: urn:nbn:nl:ui:13-6gd4-iv

Location (repository)

DANS-EASY (http://easy.dans.knaw.nl)

Deposit Date

29-7-2014

Related publications

These data underlie the paper:

Pots WTM, Meulenbeek PAM, Veehof MM, Klungers J, Bohlmeijer ET (2014). The efficacy of mindfulness-based cognitive therapy as a public mental health intervention for adults with mild to moderate depressive symptomatology: a Randomized Controlled Trial. PLOS ONE (submitted)

Subject

A community-based MBCT intervention for adults with mild to moderate depressive symptomatology.
