# Supplementary material for: The Efficacy of Mindfulness-Based Cognitive Therapy as a Public Mental Health Intervention for Adults with Mild to Moderate Depressive Symptomatology: A Randomized Controlled Trial
Source: PLoS One. 2014 Oct 15;9(10):e109789. doi: 10.1371/journal.pone.0109789 (PMC4198116; doi:10.1371/journal.pone.0109789)
Supplement: Protocol S1 — Trial protocol English translation. (PDF) [file pone.0109789.s003.pdf]

Trial protocol

A preventive mindfulness intervention

A randomised controlled trial

(English translation on main points)

Ernst Bohlmeijer
Wendy Pots

University of Twente
Faculty Behaviour Science
Postbus 217, 7500 AE, Enschede

Phone (06-51070348)

Mail e.t.bohlmeijer@gw.utwente.nl
Mail w.t.m.pots@utwente.nl
Web www.utwente.nl

Trial protocol

BACKGROUND OF THE STUDY
Depression and anxiety disorders are common health problems among adults in the Netherlands. These disorders have a major negative impact on the functioning and quality of life of the patient. Moreover, these disturbances lead to enormous health care costs annually and increased use of health services. The main risk factor for developing mental disorders is the presence of mild to moderate psychological symptoms (depression and anxiety). Interventions in the field of indicated prevention aim to reduce psychological symptoms and increase psychological flexibility thus decreasing the risk of mental disorders.

GGNet has developed a mindfulness intervention for adults with mild and moderate psychological symptoms. This intervention is based on the principles of Mindfulness Based Cognitive Therapy (MBCT; Teasdale et al., 2000). The University of Twente will perform (in cooperation with GGNet, Dimence, Mediant and GGZ Leiden) a study of the effects of the intervention. The intervention is compared to a waiting list control group. Primary outcomes are depression and anxiety. Secondary outcomes are positive mental health, psychological flexibility and mindfulness.

OBJECTIVE OF THE STUDY
The purpose of the study is to conduct a randomised controlled trial with the intervention 'Less stress by mindfulness' to study the:

1. effectiveness in terms of reduction of psychological symptoms (depression and anxiety);
2. effectiveness in terms of improvements in positive mental health, psychological flexibility and mindfulness.

The hypothesis is that the intervention group is superior to the comparison group, which offered no intervention, in terms of clinical outcomes (reduction of psychological symptoms and improvement of positive mental health, psychological flexibility and mindfulness).

The testing of the hypotheses will be unilateral, because the hypotheses are directional in nature. One-tailed tests further require a smaller sample, so that fewer subjects are needed for the study. From a medical-ethical and financial perspective this is preferable.

SCIENTIFIC EXPLANATION
Not translated.

STUDY POPULATION
Participants are adults of 18 years and older, with no apparent psychopathology. However, they have mild to moderate psychological distress.

RECRUITMENT. Participants were recruited from general population. Previous experience (e.g. the study of the group intervention 'living to the full' (Bohlmeijer et al, in press) and the intervention 'No Panic') show that recruitment is successful through advertisements in newspapers and magazines and leaflets from GPs, physiotherapists, pharmacists and libraries. The recruitment is coordinated by Ms. WTM Pots. Interested subjects will receive information in writing about the intervention and the study design. They also receive a written informed consent form. During the screening/interview, additional information on the intervention is given, explanation on study details (including

randomisation) and exclusion criteria will be checked. Diagnostic assessment is supported by part of the MINI-Plus (Sheehan et al , 1998), to exclude the presence of a serious depression. The diagnostic interview is conducted by trained staff (under the supervision of a registered psychologist). The inclusion and exclusion criteria were supervised by researcher Mrs. WTM Pots. The participants will be informed on participation in the study in writing. Randomisation then takes place. After the randomization participants receive a written information of the outcome of the randomization.

INCLUSION CRITERIA.
Adults of 18 years and older with mild to moderate psychological distress.

EXCLUSION CRITERIA
1. Serious psychopathology requiring immediate treatment measured with the M.I.N.I.-Plus. When there is a serious depression or anxiety disorder, the clients will be referred to GGNet, Dimence, Mediant or GGZ Leiden for a treatment. There has been an agreement with the Health organisations that the clients will be seen shortly (within a week).
2. People recently started on pharmacological treatment, within three months (before the start of the research). If so, it is not well deductable if the effects are to be attributed to the intervention or the pharmacological treatment.
3. Currently undergoing psychological (self-help)treatment at a mental health institution.
4. Not enough time to complete the intervention.
5. Inadequate mastery of the Dutch language (reading or learning difficulties).

MENTAL COMPETENCE. The participants were adults aged 18 and older, with no apparent psychopathology. However, these are people with mild to moderate psychological distress with an increased risk of developing mental disorders. Therefore they receive a public mental helath intervention. The adults in this intervention, which are no patients, received no treatment, but an intervention. For this reason, the participants are also considered a healthy group of subjects.

STUDY DESIGN
DESIGN. Randomized, controlled trial with two parallel groups, namely:
1. the experimental condition: the intervention 'Less stress by mindfulness' is offered.
2. the control condition: 'waiting list'/comparison group, which an intervention is offered after three months. Therefore, they are backed by a waiting list, but free to use other forms of care.

Prospective measurements with a baseline measurement and two follow-ups (at the end of the intervention and after three months). It is a pragmatic, non-blinded trial.

RANDOMIZATION. After receiving the completed informed consent forms and the results of the screening/interview, randomisation will be performed centrally at the University of Twente. Respondents are individually randomised and divided between the two conditions. In addition, stratified by sex. In this manner, there is the guarantee that the two groups with respect to gender are comparable. Participants receive in writing the outcome of the randomization.

INTERVENTION: TRAINING INTERVENTION. The mindfulness training is an intervention of 11 meetings of 1½ hours and possibly a follow-up session after 4-6 weeks, which is conducted in groups of about 8 to 15 participants. This intervention is based on MBCT. The intervention consists of three elements: attention (session 1, 2 and 3), acceptance (5, 7, 9 and 10) and dealing differently with thoughts (session 4, 6 and 8). The last meeting has evaluation as a theme. In the first sessions participants learn how they can

consciously focus their attention on the here and now. They also learn how to cope with periods of distraction from what they are doing and return to the present moment. Two basic exercises will be practised: the body scan and the focus of attention on breathing. The practice of mindfulness in daily life will be extensively dwelt on. In meetings about acceptance participants learn how they can be accept things as they are. The importance of the concepts are discussed and exercises are done to learn to accept negative emotions and thoughts. When dealing differently with thoughts, the emphasis is on learning that thoughts are not the basis of everyone's identity. One learns to observe thoughts as emerging and disappearing phenomena, distance from thoughts and not to respond to negative emotions and thoughts.
For more information see the appendix *(appendix not included in the English translation)*.

INTERVENTION: CONTROL GROUP. The control group is offered the same intervention after 3 months. They will be placed on a waiting list. The people on the waiting list are free to use other forms of care. In other recent trials within GGNet in collaboration with the University of Twente, this form of control groups is used successfully (a study on the prevention of panic disorder, 'No panic'; a study on 'Acceptance and commitment therapy' (ACT; Bohlmeijer et al, in press)). The waiting time is kept short, i.e. three months. This appears to be suitable for participants. What is relevant is that it involves people who have no serious disorder and are not seeking professional counseling.

STUDY PARAMETERS. The intervention aims to decrease psychological distress and strengthen positive mental health, psychological flexibility and mindfulness. Goals in the study are:

1. Primary: Reduction of psychological symptoms (depression, anxiety).
2. Secondary: improvement of positive mental health, psychological flexibility and mindfulness.

MEASUREMENTS.
The following table provides an overview of the instruments. All are validated instruments.

**Outcome measure**	**Measurement**	**Reference**	**Items**
Major Depressive disorder	M.I.N.I.-Plus	Sheehan e.a., 1998	39
Depression	Center for Epidemiologic Studies Depression Scale (CES-D)	Bouma e.a. 1995 Radloff (1977)	20
Anxiety	Hospital Anxiety and Depression Scale – Anxiety subscale (HADS-A)	Snaith, 2003 Zigmond & Snaith,1983.	7
Psychological flexibility	Acceptance and action questionnaire II (AAQ-II)	Jacobs et al., 2008	10
Mindfulness	Five Facet Mindfulness Questionnaire (FFMQ)	Baer et al., 2006	39
Positive mental health	Mental Health Continuum – short form (MHC-SF)	Keyes, 2005; Westerhof & Keyes, 2008	14

Demographic characteristics and other	Gender, age, education, marital status, cultural background, medication, past (psychological) treatment, time investment, mastery of the Dutch language		9
**Total**			**138**

At the end of the intervention participants will receive an evaluation form. The participants can indicate whether the intervention met their expectations, e.g. length and content, contact with the counsellors and teaching materials (difficulty level, amount of text, assignments, etc.). They can also give recommendations about the material.

MEASUREMENTS.
1. Screening ($t0^1$; de baseline)
2. Immediately prior to the intervention ($t0^2$; baseline)
3. Immediately after the intervention (t1; 3 months after baseline)
4. 6 months after baseline (t2; 3 months after the intervention)

With the exception of the demographic variables and the MINI-Plus (only at baseline $t0^1$), all instruments are taken at all three measure moments.

REPORTING the trial is carried out in accordance with the applicable international guideline: the CONSORT statement (Moher, Jones & Lepage, 2001).

TIME SCHEDULE. The study is in preparation and will run for 18 months. The study will start on January 1, 2010. If the study is approved by the METIGG, it will commence. Recruiting respondents in January 2010. Data will be collected until January 2011. The analysis and reporting will then take six months.

BURDEN AND RISKS ASSOCIATED WITH PARTICIPATION

NATURE AND EXTENT OF THE BURDEN AND RISKS *(summary in English translation)*. The subjects will attend the intervention 1½ hours weekly for 11 weeks, with possibly a follow-up session after 4 to 6 weeks, and do homework exercises 30 minutes daily. At the beginning of the study there will be a screening/meeting of 30 minutes, plus assessment of part of the M.I.N.I.-Plus of 15-30 minutes of duration. Furthermore, subjects are asked to fill in questionnaires at three times, with a load of approximately 1½-2 hours in total. We expect not many risks for the participants, because they are a relative healthy population, without psychopathology.

ADDITIONAL RISK ASSESSMENT.
We expect no risks for the participants, after all :
- Participants are self-referred because they suffer from mild to moderate mental health problems. It is a population that is reasonably healthy and function accordingly. If nevertheless there was evidence of serious psychological problems, the participants are immediately referred to the assisting community mental health centres.
- The interventions are consistently presented as opportunities to gain more control over one’s life. It is an intervention to enhance acceptance based on exercises and information.

- A possible risk may be that a participant symptoms' increase during the intervention, without them directing this to the counsellor. Intermediate questionnaires will reveal if symptoms become more severe. In the presence of an anxiety or depressive disorder, the participant is referred directly to a GP.
- In this intervention, participants are introduced to a number of themes to enhance mental health. The intervention follows a health model and not a disease model, so the risk of stigmatisation is very small.

LIMITING THE NUMBER OF SUBJECTS. The hypotheses (see page 2) are unidirectional and will therefore be tested one-sided. One-tailed tests require relatively smaller numbers of subjects (see below), which provides less ethics and more financial benefits.

STATISTICS

POWER. With a N = 51 (at t1) per condition we will have sufficient power (1 - β = 0.80) for the directional hypotheses (the difference will be in favour of the experimental condition compared to the control condition) in a one-sided test at α=0.05, the following effects are to be found: a reduction in psychological distress and improvement in positive mental health, psychological flexibility and mindfulness. Effects are expected to be at least the size from 0.50 standard units (standard effect sizes, medium impact; Lipsey, 1993). We expect that the community mental health centres will each recruit 40 participants during the study. Considering a dropout rate of 15% between t0 and t2, at least 102 (120 minus 18) participants need to be included.

ANALYSIS. The data analysis is performed using the statistical package SPSS. When analysing the following data characteristics will be taken into account: loss-to-follow-up and the nested data structure, because we are dealing with groups of subjects who receive an intervention in multiple locations. Therefore, all analyses are performed based on the intention-to-treat principle. Missing values at t1 and t2 will be imputed according to the last-observation-carried-forward principle, or in a more sophisticated way (regression imputation or multiple imputation). Second, taking into account the fact that the data have been clustered we use the first-order Tailor series linearization method by which 95% confidence intervals and test results are calculated correctly when clustered together (or "nested"). Because the hypotheses are directional in nature, the tests are one-sided at alpha=0.05 and a power of (1-beta)=0.80.

CLINICAL EVALUATION. Differences between the conditions in clinical outcomes are expressed in standardized effect sizes (d) (Lipsey & Wilson, 1993). This analysis gives clinical value of a public mental health intervention versus no intervention.

ETHICS

The researchers assumed liability for the WMO. In this context we offer the following considerations:

1. The research contributes to new insights in the field of a major, serious, costly and partly avoidable problem in public mental health (see p. 2 of the Protocol; *not included in the English translation*).
2. The intervention is new and has a positive perspective, which will be appealing to many people, with little chance of stigmatisation or reinforcement of sickness behaviour.
3. If the intervention proves to be effective, there are good chances to broadly implement the intervention in an applicable way (p. 2 of the Protocol).

4. The burden associated with participation includes participation in one interview, 11 intervention sessions and homework exercises of 30 minutes per day, plus travel time. In addition, they are asked to fill in questionnaires at three different times requiring 30 to 45 minutes. Per subject the burden is divided over 6 months (see the protocol p. 5).
5. The number of subjects is minimized, while the study has sufficient statistical power (see p. 6 of the Protocol).
6. By participating in the study, the subjects are not exposed to any known risks. All subjects (in both conditions) are at all times freely allowed to use other forms of care and to terminate their participation in the study and/or the intervention (see p. 4 of the Protocol).
7. Participation in the study is likely to provide benefits to the subjects in terms of reducing symptoms, risk of a full-blown DSM-IV Axis I mental disorder, and above all, improve their quality of life.
8. Participation in the study is voluntary with informed consent (see Appendix; *not included in the English translation*).
9. Subjects can always turn to their GP and receive care elsewhere. Under certain conditions (when there seems to be a serious problem on the basis of observation by counselors or by questionnaires) an explicit recommendation and referral for a screening at the participating community mental health centre will be advised.
10. The privacy of subjects will be guaranteed (see the protocol p. 7).

KNOWLEGDE TRANSFER AND IMPLEMENTATION
Not translated.

INSURANCE
Waiver will be requested with the reviewing ethics committee. There will be an insurance when the METIGG deems it necessary.

ANONYMITY
ANONIMITY. This is a longitudinal study with multiple measurements for the same subjects. Therefore, there can not be complete anonymity. Study data will be stored under ID code, and thus anonymous.

USE OF ID CODES. Respondents have an arbitrary ID code (which is necessary, because it involves longitudinal research).

SEPARATION OF PERSONAL AND STUDY DATA. The ID code is not traceable to the individual in question for the researchers. The assistant at the Data Collection Section (see below) has both the code and personal access, but no access to the study data. Personal data and study data will so be separated. The assistant at the Data Collection Section controls the mailing of the questionnaires, using a set up list of ID codes and addresses. The assistant transmits the (empty) questionnaires with the ID code to the respondents. Respondents will return their completed questionnaire (which has only their ID code) to the researcher. Again, personal and study data are separated, both seen from the position of assistant at the Data Collection Section as from the position of the researchers.

DESTRUCTION OF PERSONAL DATA. Within a span of six (at most seven) months, the study data will be collected. From that moment on, there is no need to preserve the

personal data. However, the ID codes of the study will be stored to ensure that any (legal) control of the data remains possible for 15 years. The aforementioned list of personal data and the ID codes (thus far in possession of the Assistant Data Collection) will be destroyed by the responsible lead researcher, Dr. Ernst Bohlmeijer. From then, only anonymous study data are available with an obligation to retain.

INSPECTION. Subjects have the right to inspect their collected data. They can submit a written request to the responsible researcher, Dr. Ernst Bohlmeijer. The request does not need to be motivated and will always be met. Each participant will receive information at the beginning of this study. The right to inspect cannot be met once the personal data is destroyed. The study data is then anonymous.

CODE OF GOOD BEHAVIOR. Both researchers and the assistant at the Department of Data Collection are bound by the Code of Good Conduct, which is an elaboration of the privacy legislation.

PROJECTGROEP

Dr. E. Bohlmeijer is associate professor at the University of Twente.

Drs. J. Klungers is manager at GGNet, Community Mental Health Centre, and adapted the intervention for purposes of their population.

Drs. W.T.M. Pots is assistant professor at the University of Twente and clinical psychologist at Dimence, Community Mental Health Centre.

REFERENCES

Baer, R.A. (2003). Mindfulness training as a clinical intervention: A conceptual and empirical review. *Clinical Psychology: Science and Practice, 10*(2), 125-143.

Baer, R.A., Smith, G.T., Hopkins, J., Krietemeyer, J., & Toney, L. (2006). Using self-report assessment methods to explore facets of mindfulness. *Assessment, 13*(1), 27-45.

Beekman, A.T.F., Cuijpers, P., Marwijk, H.W.J. van, Smit, F., Schoevers, R.A & Hosman, C. (2006). Preventie van psychiatrische stoornissen. *Nederlands Tijdschrift voor de Geneeskunde, 150*, 419- 423

Bijl, R.V., Ravelli, A., & Van Zessen, G. (1998). Prevalence of psychiatric disorder in the general population: Results of the Netherlands Mental Health Survey and Incidence Study (NEMESIS). *Social Psychiatry and Psychiatric Epidemiology, 33*(12), 587-595.

Bohlmeijer, E. T, Fledderus, M., Pieterse, M.E. (in voorbereiding). The effects of acceptance and commitment therapy as an early intervention on psychological distress in adults: results of a pilot RCT.

Bouma, J., Ranchor, A.V., Sandersman, R. & Van Sonderen, F.L.P. (1995). *Het meten van symptomen van depressie met de CES-D. Een handleiding.* Noordelijk Centrum voor Gezondheidsvraagstukken, Rijksuniversiteit Groningen.

Hayes, S.C. , Luoma, J.B., Bond, F.W., Masuda, A. & Lillis, J. (2006). Acceptance and Commitment Therapy: Model, processes and outcomes. *Behaviour Research and Therapy, 44,* 1-25.

Jacobs, N., Kleen, M., Blokzijl, R., Groot, F. de & A-Tjak, J. (2008). Measuring experiential avoidance. Dutch translation of the Acceptance and Action questionnaire-II (AAQ-II). *Gedragstherapie, 41*, 349-361.

Kabat-Zinn, J. (1990). *Full catastrophe living: Using the wisdom of your body and mind to face stress, pain and illness.* New York: Delacorte.

Keyes, C.L.M. (2005). Mental Illness and/or Mental Health? Investigating Axioms of the Complete State Model of Health. *Journal of Consulting and Clinical Psychology*, *73*(3), 539–548.

Keyes, C.L.M. (2007). Promoting and Protecting Mental Health as Flourishing A Complementary Strategy for Improving National Mental Health. *American Psychologist, 95*(62), 95–108.

Ma, S.H., & Teasdale, J.D. (2004). Mindfulness-Based Cognitive Therapy for Depression: Replication and Exploration of Differential Relapse Prevention Effects. *Journal of Consulting and Clinical Psychology, 72*(1), 31-40.

Meijer, S.A., Smit, F., Schoemaker, C.G. & Cuijpers, P. (2006). *Gezond verstand, evidence-based preventie van psychische stoornissen.* Bilthoven: RIVM.

Moher, D., Jones, A. & Lepage, L. (2001). Use of the CONSORT Statement and Quality of Reports of Randomized Trials. A comparative Before-and-After Evaluation. *Journal of the Americal Midcal Association, 285,* 1992-1995.

Lipsey, M.W. (1990). *Design sensitivity: statistical power for experimental research*. New York: Sage.

Lipsey, M. W., & Wilson, D. B. (1993). The efficacy of psychological, educational, and behavioral treatment: Confirmation from meta-analysis. *American Psychologist, 48,* 1181-1209.

Orsillo, S.M., Roemer, L., & Barlow, D.H. (2003). Integrating Acceptance and Mindfulness into Existing Cognitive-Behavioral Treatment for GAD: A Case Study. *Cognitive and Behavioral Practice, 10*(3), 222-230.

Radloff, L.S. (1977). The CES-D scale: A self-report depression scale for research in the general population. *Applied Psychological Measurement*, 1, 385–401.

Roemer, L., Orsillo, S.M., & Salters-Pedneault, K. (2008). Efficacy of an Acceptance-Based Behavior Therapy for Generalized Anxiety Disorder: Evaluation in a Randomized Controlled Trial. *Journal of Consulting and Clinical Psychology, 76*(6), 1083-1089.

Roemer, L., Lee, J.K., Salters-Pedneault, K., Erisman, S.M., Orsillo, S.M., & Mennin, D.S. (2009). Mindfulness and Emotion Regulation Difficulties in Generalized Anxiety Disorder: Preliminary Evidence for Independent and Overlapping Contributions. *Behavior Therapy, 40*(2), 142-154.

Sheehan. D.V., Janavs, J., Baker R, Harnett-Sheehan, K., Knapp, E., Scheehan, M.F. & Dunbar, G.C. (1998). M.I.N.I. – Mini International Neuropsychiatric Interview- English version 5.0.0. – DSM-IV. *Journal of Clinical psychiatry, 59*, 34-57.

Smit, F., Beekman, A., Cuijpers, P. , De Graaf, R. & Vollebergh, W. (2004). Selecting key variables for depression pervention: results from a population-based prospective epidemiological study. *Journal of Affective Disorders, 81*, 241-249.

Snaith, R.P., (2003) The Hospital Anxiety And Depression Scale. *Health and Quality of*

Life Outcomes, 1-29.

Teasdale, J. D., Segal, Z.V., Williams, J.M., Ridgeway, V.A., Soulsby, J.M. & Lau, M.A. (2000). Prevention of relapse/recurrence in major depression by mindfulness-based cognitive therapy. *Journal of Consulting and Clinical Psychology, 68(4),* 615-23.

Westerhof, G.J. & Keyes, C.L.M. (2008). Geestelijke gezondheid is meer dan de afwezigheid van geestelijke ziekte. *Maandblad Geestelijke Gezondheidszorg, 10*(63), 808-820.

Zigmond, A.S. & Snaith, R.P. (1983). The Hospital Anxiety and Depression Scale. *Acta Psychiatrica Scandinavica, 67*, 361–370.
