# Supplementary material for: The Efficacy of Mindfulness-Based Cognitive Therapy as a Public Mental Health Intervention for Adults with Mild to Moderate Depressive Symptomatology: A Randomized Controlled Trial
Source: PLoS One. 2014 Oct 15;9(10):e109789. doi: 10.1371/journal.pone.0109789 (PMC4198116; doi:10.1371/journal.pone.0109789)
Supplement: Protocol S2 — Trial protocol original Dutch language. (PDF) [file pone.0109789.s004.pdf]

Onderzoeksprotocol

Mindfulness preventie

Een gerandomiseerd gecontroleerd onderzoek

Penvoerder

Ernst Bohlmeijer
Wendy Pots

Universiteit Twente
Faculteit Gedragswetenschappen
Gebouw Citadel, kamer H401
Postbus 217, 7500 AE, Enschede

Tel. (06-51070348)

Mail e.t.bohlmeijer@gw.utwente.nl
Web www.utwente.nl

Onderzoeksprotocol

SAMENVATTING

Depressie en angststoornissen zijn veel voorkomende gezondheidsproblemen onder volwassenen in Nederland. Deze stoornissen hebben een grote negatieve invloed op het functioneren en de kwaliteit van leven van de patiënt. Bovendien leiden dergelijke stoornissen jaarlijks tot enorme zorgkosten. De belangrijkste risicofactor voor het ontwikkelen van psychische stoornissen is de aanwezigheid van matige depressieve en angstklachten. Geïndiceerde preventie heeft als doel de reductie van psychische klachten en het vergroten van de psychologische flexibiliteit waardoor de kans op psychische stoornissen afneemt.

GGNet heeft een preventieve mindfulness training ontwikkeld voor volwassenen met lichte en matige psychische klachten. Deze cursus is gebaseerd op de uitgangspunten en principes van Mindfulness Based Cognitive Therapy (MBCT). De Universiteit Twente zal in samenwerking met GGNet, Dimence, Mediant en GGZ Leiden een onderzoek uitvoeren naar de effecten van de cursus. De cursus wordt vergeleken met een wachtlijstcontrolegroep. Primaire uitkomsten zijn angst en depressie. Secundaire uitkomsten zijn positieve geestelijke gezondheid, experiëntiële vermijding/psychologische flexibiliteit en mindfulness.

VRAAGSTELLING

DOELEN van het voorgestelde onderzoek zijn de cursus 'Minder stress door aandacht' empirisch te toetsen op

1. de effectiviteit in termen van afname van psychische klachten (depressieve klachten en angstklachten);
2. de effectiviteit in termen van verbetering in positieve geestelijke gezondheid, psychologische flexibiliteit en mindfulness

HYPOTHESEN.
De interventiegroep is superieur aan een controlegroep, die geen preventieve interventie krijgt aangeboden, in termen van de klinische uitkomsten (vermindering van psychische klachten en verbetering van positieve geestelijke gezondheid, psychologische flexibiliteit, mindfulness).

De toetsing van de hypothesen zal eenzijdig plaatsvinden, omdat de hypothesen directioneel van aard zijn. Een eenzijdige toets vergt verder een kleinere steekproef, zodat ook minder proefpersonen voor het onderzoek nodig zijn. Vanuit medisch-ethisch en financieel perspectief heeft dit de voorkeur.

WETENSCHAPPELIJKE VERANTWOORDING

EPIDEMIOLOGISCHE ONDERBOUWING. Depressie en angststoornissen komen veel voor en vormen een ernstig gezondheidsprobleem. Jaarlijks lijdt 1 op de 4 Nederlanders aan één van deze stoornissen (Meijer e.a., 2006). Ze behoren tot de top-10 van ziekten met de grootste ziektelast. Depressie heeft de hoogste prevalentie bij volwassenen. Jaarlijks hebben ongeveer 600.000 volwassenen in Nederland te kampen met een depressie (Bijl, Ravelli, & Van Zessen, 1998). Preventie kan een belangrijke rol spelen bij het voorkomen

van de instroom van nieuwe gevallen en het terugdringen van psychische klachten bij volwassenen. Verreweg de belangrijkste risicofactor voor het krijgen van een psychische stoornis is het hebben van lichte tot matige psychische klachten (Smit e.a., 2004). Er is dus een grote behoefte aan laagdrempelige, preventieve interventies voor volwassenen die leiden tot een afname van psychische klachten en daarmee tot een reductie in de incidentie van het ontwikkelen van een psychische stoornis (Beekman et al., 2006).

VERMOEDELIJKE EFFECTIVITEIT. Mindfulness is een dergelijke laagdrempelige methodiek. Mindfulness is gedefinieerd als het intentioneel en accepterend aandacht hebben voor wat er in het nu is (Kabat-Zinn, 1990). De kern van mindfulness training is meditatie. In eerste instantie werd een programma ontwikkeld voor het leren omgaan met stress bij mensen met chronische ziekten: Mindfulness Based Stress Reduction (MBSR). Later werd MBSR geïntegreerd met cognitieve gedragstherapie, het zogenaamde Mindfulness Based Cognitive Therapy (MBCT). Meer dan in MBSR wordt aandacht besteed aan het leren omgaan met negatieve gedachten en emoties. Het verschil met cognitieve therapie is dat niet wordt ingegaan op de inhoud van gedachten. Er wordt een metacognitief bewustzijn ontwikkeld. Men leert gedachten observeren als opkomende en verdwijnende fenomenen. Hierdoor wordt geleerd om niet te reageren op negatieve emoties en gedachten waardoor de invloed op het feitelijke gedrag wordt verminderd. MBCT is zeer effectief gebleken bij mensen met recidiverende depressies (Ma & Teasdale, 2004; Teasdale, et al., 2000). Inmiddels zijn ook eerste onderzoeken gedaan naar de toepassing bij mensen met een depressie of angststoornis zonder dat er sprake is van recidive. De resultaten zijn veelbelovend (Baer, 2003; Orsillo, Roemer, & Barlow, 2003; Roemer, et al., 2009; Roemer, Orsillo, & Salters-Pedneault, 2008).
Hoewel MBCT ook in de preventieve geestelijke gezondheidszorg wordt toegepast, is er nog geen onderzoek gedaan naar de effectiviteit. In dit onderzoek zal het preventieprogramma, gebaseerd op MBCT en door GGNet ontwikkeld, op effectiviteit worden onderzocht. De cursus is al verscheidene malen uitgevoerd en deelnemers zijn erg enthousiast over het programma.

RELEVANTIE

1. RELEVANTIE: EVIDENCE-BASED. Binnen de (preventieve) geestelijke gezondheidszorg wordt veel waarde gehecht aan onderzoek naar de effectiviteit van interventies. Gerandomiseerd effectonderzoek wordt daarbij als geprefereerde methode gezien om de effectiviteit aan te tonen.

2. RELEVANTIE: IMPLEMENTATIEKANSEN. Er is binnen de geestelijke gezondheidszorg veel belangstelling voor cursussen gericht op preventie van psychische stoornissen. Zo worden mindfulness trainingen in toenemende mate vanuit preventie aangeboden. Onderbouwing van de effectiviteit van mindfulness is een belangrijke voorwaarde voor verdere implementatie.

RELEVANTIE: INNOVATIE. Volwassenen met lichte tot matige psychische klachten zijn voor preventie een belangrijke doelgroep. Zij worden op dit moment echter slecht bereikt met bestaande interventies (Meijer et al., 2006). Een van de oplossingen om het bereik te vergroten is om ten eerste preventieve interventies van een positief kader aan te bieden. De verwachting is dat preventie daarmee aantrekkelijker en laagdrempelig wordt. Er wordt geworven onder het motto van het versterken van de geestelijke gezondheid en psychologische flexibiliteit, in plaats van de nadruk te leggen op het verminderen van psychische klachten. Versterking van de psychologische flexibiliteit en geestelijke gezondheid beschermt tegen psychische klachten en stoornissen (Hayes et al., 2006; Westerhof & Keyes, 2008; Keyes, 2007). Ten tweede trekt de cursus 'Minder

stress door aandacht', in tegenstelling tot veel bestaande trainingen, veel belangstelling. Er kunnen mensen met verschillende psychische klachten deelnemen, waardoor de interventie zich niet beperkt tot één bepaalde psychische klacht.

DOELGROEP

De doelgroep bestaat uit volwassenen van 18 jaar en ouder die last hebben van lichte tot matige psychische klachten (depressieve klachten en angstklachten) en/of mensen met een lage geestelijke gezondheid (selectieve preventie, de afwezigheid van geestelijke gezondheid maakt mensen kwetsbaar voor psychische klachten).

WERVING. Er wordt gebruik gemaakt van een open werving. Eerdere ervaringen (bijvoorbeeld het onderzoek naar de cursus Geen Paniek en de cursus Voluit Leven) laten zien dat de werving goed verloopt via advertenties in kranten en tijdschriften en via informatiefolders bij huisartsen, fysiotherapeuten, apothekers en bibliotheken. De werving wordt gecoördineerd door mevrouw W.T.M. Pots. Belangstellenden krijgen schriftelijk een informatiebrief over de cursus en over de onderzoeksopzet. Bij de informatie ontvangen zij tevens schriftelijk een informed-consent formulier. Tijdens het kennismakingsgesprek wordt extra informatie gegeven over de cursus, wordt uitgelegd wat het onderzoek inhoudt (inclusief de randomisatie) en wordt nagegaan of er sprake is van exclusiecriteria. De diagnostiek wordt ondersteund door gedeeltelijk het diagnostisch interview de M.I.N.I.-Plus (Sheehan e.a., 1998) af te nemen, om de aanwezigheid van een depressieve stoornis en/of angststoornis uit te sluiten. Het diagnostisch interview wordt afgenomen door getrainde medewerkers (onder supervisie van een BIG geregistreerde GZ-psycholoog). De beoordeling van de M.I.N.I-Plus om te bepalen of de deelnemer voldoet aan de in- en exclusiecriteria wordt uitgevoerd door onderzoeker mevrouw W.T.M. Pots. Vervolgens krijgen de deelnemers schriftelijk de uitslag of zij mogen deelnemen aan het onderzoek of niet. Vervolgens vindt de randomisatie plaats. De deelnemers ontvangen schriftelijk de uitkomst van de randomisatie.

INCLUSIECRITERIA. De doelgroep voor deze studie zijn volwassenen die last hebben van psychische klachten (angstklachten en/of depressieve klachten).

EXCLUSIECRITERIA

Als exclusiecriteria hanteren we (in navolging van de bestaande praktijk):

1. De aanwezigheid van ernstige psychopathologie. Wanneer er sprake is van een ernstige depressieve en/of angststoornis volgens de M.I.N.I-Plus worden de cliënten verwezen voor een intakegesprek van GGNET, Dimence, Mediant of GGZ Leiden. Met de GGZ-instellingen is de afspraak gemaakt dat de cliënt op korte termijn (binnen een week) in het kader van een intake voor behandeling wordt gezien.
2. Mensen die in de drie maanden voor aanmelding zijn gestart met medicatie in verband met hun klachten. Wanneer dit het geval is, is het niet goed mogelijk te onderscheiden of effecten aan de medicatie of aan de cursus kunnen worden toegeschreven.
3. Het ontvangen van een lopende psychologische (zelfhulp)behandeling.
4. Niet voldoende tijd om de cursus goed te volgen.
5. Slechte beheersing van de Nederlandse taal (lees- of leermoeilijkheden).

WILSBEKWAAMHEID. De deelnemers zijn volwassen personen van 18 jaar en ouder, zonder duidelijke psychopathologie. Wel zijn het personen met lichte tot matige psychische klachten. Deze personen hebben hiermee een verhoogd risico een klinische psychische stoornis te ontwikkelen. Daarom krijgen zij een vroegtijdige interventie

aangeboden. De volwassenen in deze interventie, die geen patiënten zijn, ontvangen dus geen behandeling, maar een cursus. Om deze reden wordt de groep deelnemers dan ook beschouwd als een gezonde groep proefpersonen.

ONDERZOEKSOPZET

DESIGN. Gerandomiseerde, gecontroleerde studie met twee parallelle groepen, namelijk
1. de experimentele conditie: de cursus 'Minder stress door aandacht'
2. de controlegroep: 'wachtlijst' vergelijkingsgroep.

Prospectieve metingen met één baseline meting en twee follow-ups (direct aan het einde van de cursus en na drie maanden). Het betreft een pragmatische, niet-geblindeerde trial.

RANDOMISATIE. Na het ontvangen van de ingevulde 'informed consent' formulieren en de uitkomsten van het intakegesprek/interview, zal de randomisatie centraal uitgevoerd worden op de Universiteit Twente. Respondenten worden individueel gerandomiseerd en verdeeld over de twee condities. Daarbij wordt gestratificeerd naar geslacht. Op deze wijze is er de garantie dat de twee groepen ten aanzien van geslacht optimaal te vergelijken zijn. De deelnemers ontvangen schriftelijk de uitkomst van de randomisatie.

CONDITIES: DE CURSUS. De cursus 'Minder stress door aandacht' is een groepsgerichte interventie van 11 bijeenkomsten van 1½ uur en mogelijk een follow-up bijeenkomst na 4-6 weken, die wordt uitgevoerd in groepen van ongeveer 8-15 deelnemers. Deze interventie is gebaseerd op MBCT. De training bestaat uit drie onderdelen: aandacht (bijeenkomst 1, 2 en 3), acceptatie (5, 7, 9 en 10) en anders omgaan met gedachten (bijeenkomst 4, 6 en 8). De laatste bijeenkomst heeft als thema evaluatie. In de eerste bijeenkomsten leren de deelnemers hoe ze bewust hun aandacht kunnen richten op het hier en nu. Ook leren ze hoe ze kunnen omgaan met momenten waarbij ze afgeleid raken van datgene waar ze mee bezig zijn en weer terug kunnen keren naar dit moment. Er wordt geoefend met twee basisoefeningen: de lichaamsscan en het richten van de aandacht op de ademhaling. Er wordt uitgebreid stilgestaan bij het beoefenen van aandacht in het dagelijks leven. In de bijeenkomsten rondom acceptatie leren deelnemers hoe ze situaties kunnen accepteren zonder deze te willen veranderen. Er wordt stilgestaan bij de betekenis van de begrippen en er worden aandachtsoefeningen gedaan om negatieve emoties en gedachten te leren accepteren. Bij anders omgaan met gedachten ligt de nadruk op het leren dat gedachten niet de basis zijn van ieders identiteit. Men leert gedachten observeren als opkomende en verdwijnende fenomenen, afstand te nemen van gedachten en niet te reageren op negatieve emoties en gedachten. Voor meer informatie zie de appendix.

CONDITIES: CONTROLEGROEP. De deelnemers in de controleconditie ontvangen in eerste instantie geen interventie. Wel wordt hen aangeboden om na drie maanden de training te volgen. Zij komen daarvoor op een wachtlijst te staan. Het staat de mensen op de wachtlijst uiteraard vrij om gebruik te maken van andere vormen van zorg. Ook in recente andere trials binnen GGNet in samenwerking met de Universiteit Twente is deze vorm van controle groepen met succes toegepast (onderzoek naar preventie van paniekstoornissen, Geen Paniek; onderzoek naar Acceptance and commitment therapy (ACT)). De wachttijd wordt kort gehouden, namelijk drie maanden. In de praktijk blijkt dit op geen bezwaren te stuiten bij deelnemers. Relevant is dat het om mensen gaat die geen ernstige klachten hebben en ook niet uit zichzelf hulp hebben gezocht.

EINDTERMEN. De interventie is gericht op afname van psychische klachten en het versterken van de positieve geestelijke gezondheid, psychologische flexibiliteit en mindfulness. Als eindtermen gelden in deze studie:

1. Primair: Vermindering van psychische klachten (depressieve klachten en angstklachten).
2. Secundair: verbetering van positieve geestelijke gezondheid.

MEETINSTRUMENTEN.

In onderstaande tabel staat een overzicht van de meetinstrumenten. Het betreft in alle gevallen gevalideerde meetinstrumenten.

**Uitkomstmaat**	**Meetinstrument**	**Referentie**	**Aantal items**
Depressieve stoornis en angststoornis	M.I.N.I.-Plus interview	Sheehan e.a., 1998	39
Depressie	Center for Epidemiologic Studies Depression Scale (CES-D)	Bouma e.a. 1995 Radloff (1977)	20
Angst	Hads-A	Snaith, 2003 Zigmond & Snaith,1983.	7
Psychologische flexibiliteit	Acceptance and action questionnaire II (AAQ-II)	Jacobs et al., 2008	10
Mindfulness	Five Facet Mindfulness Questionnaire (FFMQ)	Baer et al., 2006	39
Positieve geestelijke gezondheid	Mental Health Continuum – short form (MHC-SF)	Keyes, 2005; Westerhof & Keyes, 2008	14
Demografische variabelen en overig	Geslacht, leeftijd, opleiding, burgerlijke staat, culturele achtergrond, medicatie, eerdere (psychologische) behandeling, tijdsinvestering, beheersing van de Nederlandse taal.		9
**Totaal**			**138**

Aan het eind van de cursus krijgen de deelnemers een evaluatieformulier. Hierbij kunnen de deelnemers aangegeven of de cursus voldeed aan hun verwachtingen, wat ze vonden van (de duur en inhoud) het contact met de begeleiders en het lesmateriaal (moeilijkheidsgraad, hoeveelheid tekst, opdrachten enz.). Ook kunnen zij hier aanbevelingen geven over het materiaal.

MEETMOMENTEN.

1. Bij kennismakingsgesprek ($t0^1$; de baselinemeting)
2. Direct voorafgaand aan de interventie ($t0^2$; de baselinemeting)
3. Direct na afloop van de interventie (t1; 3 maanden na de baselinemeting)
4. 6 maanden na de baselinemeting (t2; 3 maanden na afloop van de cursus)

Met uitzondering van de demografische variabelen en de M.I.N.I.-Plus (alleen bij de baseline $t0^1$) worden alle meetinstrumenten op alle drie de meetmomenten afgenomen.

RAPPORTAGE van de trial geschiedt conform de daarvoor geldende, internationale richtlijn: de CONSORT statement (Moher, Jones & Lepage, 2001).

TIJDPAD: Het onderzoek is in voorbereiding en heeft een looptijd van 18 maanden. Het onderzoek zal starten op 1 januari 2010. Wanneer het onderzoek wordt goedgekeurd door de METIGG, zal in januari 2010 met de werving van respondenten worden begonnen. De dataverzameling vindt plaats tot januari 2011. De analyse en verslaglegging zullen hierna 6 maanden in beslag nemen.

BELASTING VOOR DE PROEFPERSONEN

BELASTING VOORTVLOEIEND UIT DE CURSUS. De cursus bestaat uit 11 cursusbijeenkomsten (à 1½ uur per bijeenkomst) en mogelijk een follow-up bijeenkomst na 4-6 weken, die wordt uitgevoerd in groepen van ongeveer 8 tot 15 deelnemers. Daarnaast dienen de proefpersonen bereid te zijn om gedurende de cursus dagelijks 30 minuten zelfstandig bezig te zijn met opdrachten uit de training.
Voor het volgen van de cursus zal een kleine afstand moeten worden gereisd.

BELASTING VOORVLOEIEND UIT HET ONDERZOEK.
Experimentele condities: De proefpersonen krijgen aan het begin voor deelname een kennismakinggesprek van 30 minuten aangeboden. Daarnaast krijgen zij een korte lijst aangeboden met enkele demografische variabelen en zal de M.I.N.I.-Plus gedeeltelijk worden afgenomen. Alleen de diagnostische modules depressieve stoornis en angststoornissen van de M.I.N.I.-Plus worden hierbij afgenomen. Het beantwoorden van deze modules van de M.I.N.I.-Plus neemt ongeveer 15-30 minuten in beslag. Dit geeft een totale belasting van ongeveer 45-60 minuten ($t0^1$). Verder vullen ze voor het onderzoek vragenlijsten in op drie momenten; voorafgaand aan de interventie ($t0^2$), direct na de interventie (t1) en 3 maanden na beëindiging van de interventie (t2). Het invullen van de vragenlijsten op $t0^2$, t1 en t2 zal elk ongeveer 30 minuten in beslag nemen.
De totale belasting uit het onderzoek voor de proefpersonen in de experimentele conditie is dan 135-150 minuten vragenlijsten.

Controleconditie: De proefpersonen krijgen aan het begin voor deelname een kennismakinggesprek van 30 minuten aangeboden. Daarnaast krijgen zij een korte lijst aangeboden met enkele demografische variabelen en zal de M.I.N.I.-Plus gedeeltelijk worden afgenomen. Alleen de diagnostische modules depressieve stoornis en angststoornissen van de M.I.N.I.-Plus worden hierbij afgenomen. Het beantwoorden van deze modules van de M.I.N.I.-Plus neemt ongeveer 15-30 minuten in beslag. Dit geeft een totale belasting van ongeveer 45-60 minuten ($t0^1$). Verder vullen ze voor het onderzoek vragenlijsten in op drie momenten; voorafgaand aan de interventie ($t0^2$), direct na de interventie (t1) en 3 maanden na beëindiging van de interventie (t2). Het invullen van de vragenlijsten op $t0^2$, t1 en t2 zal elk ongeveer 30 minuten in beslag nemen.

De totale belasting uit het onderzoek voor de proefpersonen in de controleconditie is dan 135-150 minuten vragenlijsten.

AANVULLENDE TAXATIE VAN RISICO'S:
Wij verwachten voor de proefpersonen geen risico's. Immers:
- Proefpersonen melden zich uit eigen beweging aan omdat ze last hebben van lichte tot matige psychische klachten. Het betreft een populatie die redelijk gezond en goed functioneert. Wanneer er toch sprake mocht blijken van ernstige psychische problematiek wordt cliënt direct verwezen voor een intake bij de betreffende GGZ-instellingen.
- De interventies worden te allen tijde en consequent gepresenteerd als mogelijkheden om meer greep op het eigen leven te krijgen. Het gaat om een cursus om aan de hand van oefeningen de acceptatie trachten te vergroten.
- Een mogelijk risico kan zijn dat de klachten van een deelnemer tijdens de interventie ernstiger worden, maar dat hij of zij dit niet aangeeft bij de counselor. Doordat er vragenlijsten tussendoor worden afgenomen, kan worden bekeken of de klachten ernstiger zijn geworden. Bij de aanwezigheid van een angststoornis of depressieve stoornis wordt de deelnemer direct verwezen naar de huisarts.
- In deze interventie worden de deelnemers uitgenodigd om aan de hand van een aantal thema's de geestelijke gezondheid te versterken. De cursus volgt een gezondheidsmodel en niet een ziektemodel. De kans op stigmatisatie is daarmee zeer gering.

BEPERKING VAN HET AANTAL PROEFPERSONEN. De te toetsen hypothesen (zie pagina 2) zijn unidirectioneel en zullen daarom 1-zijdig getoetst worden. Eenzijdige toetsing vraagt om relatief kleinere aantallen proefpersonen (zie beneden), en dat biedt medisch-ethische en financiële voordelen.

STATISTIEK

POWER. Met N= 51 (op t1) per conditie wordt beschikt over voldoende power ($1-\beta=0.80$) om de directionele hypothesen (die meerwaarde verwachten van de experimentele conditie t.o.v. de controleconditie) in een 1-zijdige toets bij $\alpha=0.05$ de volgende effecten als significant in de statistieken terug te vinden: een reductie in psychische klachten en een verbetering van positieve geestelijke gezondheid, psychologische flexibiliteit en mindfulness. Effecten die worden verwacht zijn ten minste ter grootte van 0.50 standaard eenheden (standaard effectgroottes, middelgroot effect; Lipsey, 1993). Wij verwachten dat de instellingen ieder 40 deelnemers zal werven gedurende het onderzoek. Rekening houdend met een drop-out percentage van 15% tussen t0 en t2 blijven minimaal 102 (120 minus 18) deelnemers geïncludeerd.

ANALYSE. De data-analyse wordt uitgevoerd met behulp van het statistisch pakket SPSS. Bij de analyse zal met (ten minste) twee datakarakteristieken rekening worden gehouden: loss-to-follow-up en de geneste datastructuur omdat we hier te maken hebben met groepen proefpersonen die op meerdere locaties een cursus ontvangen. Daarom worden alle analyses ten eerste uitgevoerd op basis van het 'intention-to-treat' principe. Wanneer, ten gevolge van uitval, op t1 en t2 missende waarden zijn ontstaan op de klinische en/of economische eindtermen dan worden die geïmputeerd volgens het last-observation-carried-forward principe, of op een meer geavanceerde manier (regression imputation of multiple imputation). Ten tweede wordt rekening gehouden met het feit dat de data geclusterd zijn. Daarom wordt gebruik gemaakt van de eerste-orde Tailor-series linearisatie methode waarmee 95% betrouwbaarheidsintervallen en toetsuitslagen correct berekend worden wanneer data geclusterd (of 'genest') zijn.

Omdat de hypothesen directioneel van aard zijn, worden de toetsen 1-zijdig bij alfa=0.05 en een power van (1-beta)=0.80 uitgevoerd.

KLINISCHE EVALUATIE: Verschillen tussen de condities in klinische uitkomsten worden uitgedrukt in gestandaardiseerde effectgroottes (d) (Lipsey & Wilson, 1993). Met deze analyses wordt zicht gekregen op de klinische meerwaarde van preventieve cursus ten opzichte van geen preventie.

ETHISCHE OVERWEGINGEN

De onderzoekers zijn er vanuit gegaan dat het onderzoek WMO-plichtig is. In dat kader bieden wij ter toetsing de volgende overwegingen:

1. Het onderzoek levert een bijdrage aan nieuwe inzichten op het gebied van een omvangrijk, ernstig, kostbaar en deels vermijdbaar probleem in de volksgezondheid (zie p. 2 van het protocol).
2. Het betreft een nieuw, positief preventieaanbod dat voor veel mensen aantrekkelijk zal zijn met geringe kans op stigmatisatie of versterking van de ziekterol.
3. Als de interventie bewezen effectief blijkt te zijn, dan zijn er goede kansen om de interventie breed te implementeren en breed aan te bieden (p. 3 van het protocol).
4. De belasting van het onderzoek voor de proefpersonen bestaat uit deelname aan 1 kennismakingsgesprek, 11 cursusbijeenkomsten en zelfwerkzaamheid van 30 minuten per dag, plus reistijd. Daarnaast worden zij op drie verschillende momenten gevraagd vragenlijsten in te vullen. Per keer vergt dat 30 a 45 minuten. Per proefpersoon is één en ander is verdeeld over 6 maanden (zie p. 7 van het protocol).
5. Het aantal proefpersonen is zo gering mogelijk gehouden, terwijl de studie wel over voldoende power (onderscheidend vermogen in de statistische toetsuitslagen) beschikt (zie p. 8 van het protocol).
6. Door deelname aan het onderzoek lopen de proefpersonen geen bekende of noemenswaardige risico's. Het staat alle proefpersonen (in beide condities) op alle momenten vrij om elders van andere hulp gebruik te maken en om hun deelname aan het onderzoek en/of aan de cursus te beëindigen (zie p. 8 van het protocol).
7. Deelname aan het onderzoek zal naar alle waarschijnlijkheid voordelen bieden aan de proefpersonen in termen van klachtenvermindering, risicoreductie (op het ontstaan van een full-blown DSM-IV as-I psychische stoornis) en vooral meer algemeen, verbetering van hun kwaliteit van leven.
8. Proefpersonen nemen alleen deel aan het onderzoek op basis van vrijwilligheid en informed consent (zie bijlage).
9. Proefpersonen kunnen zich te allen tijde tot hun huisarts wenden of elders zorg ontvangen. Onder bepaalde omstandigheden (wanneer op basis van observatie door de begeleiders of via de vragenlijsten er sprake lijkt te zijn van ernstige problematiek) zal hen dat ook expliciet aangeraden worden en kan verwezen worden voor een intakegesprek bij de deelnemende instellingen.
10. De privacy van proefpersonen wordt naar vermogen gegarandeerd (zie p. 10 van het protocol).

KENNISOVERDRACHT EN IMPLEMENTATIE

GGNet zal in samenwerking met de Universiteit Twente in ieder geval een artikel over het onderzoek schrijven in een veel gelezen tijdschrift binnen de geestelijke gezondheidszorg. Daarnaast kunnen de resultaten gepresenteerd worden op congressen.

Er zal een studiedag worden georganiseerd waarop de resultaten bekend worden gemaakt.
De resultaten worden bekend gemaakt op het platform angst en depressie van het Trimbos-instituut.

VERZEKERING
Er wordt aan de oordelende toetsingscommissie ontheffing gevraagd. De proefpersonen zullen worden verzekerd wanneer de METIGG dit noodzakelijk acht.

INZAGERECHT EN ANONIMITEIT

ANONIMITEIT. Het betreft longitudinaal onderzoek met meerdere metingen bij dezelfde proefpersonen. Daarom kan het onderzoek niet uitgevoerd worden op basis van anonimiteit. Onderzoeksgegevens worden onder ID-code opgeslagen, en aldus geanonimiseerd.

GEBRUIK VAN ID-CODES. Respondenten krijgen een eigen arbitraire ID-code (dat is nodig, want het betreft longitudinaal onderzoek).

SCHEIDING VAN ONDERZOEKSGEGEVENS EN PERSOONSGEGEVENS. De ID-code is voor de onderzoekers niet herleidbaar tot de persoon in kwestie. Degene die zowel tot de code als de persoonsgegevens toegang heeft (assistent op de afdeling Dataverzameling, zie beneden), heeft geen toegang tot de onderzoeksgegevens. Zo blijven, vanuit de onderzoekers bezien, persoonsgegevens en onderzoeksgegevens gescheiden.
De assistent op de afdeling Dataverzameling regelt de mailing van de vragenlijsten. Die assistent heeft daartoe een lijst met ID-codes en adressen, en verzendt daarmee de (niet ingevulde) vragenlijsten (voorzien van de ID-code) naar de respondenten. De respondenten sturen de ingevulde vragenlijst (waarop alleen hun ID-code staat) rechtstreeks terug aan de onderzoeker. Zo blijven opnieuw persoonsgegevens en onderzoeksgegevens gescheiden, zowel bezien vanuit de positie van de assistent op de afdeling Dataverzameling als die van de onderzoekers.

VERNIETIGING VAN PERSOONSGEGEVENS. Binnen de tijdsspanne van zes (hooguit zeven) maanden zullen de onderzoeksgegevens verzameld zijn. Vanaf dat moment vervalt de noodzaak om de persoonsgegevens te bewaren. Wel zullen de ID-codes van het onderzoek 15 jaar bewaard moeten blijven opdat eventuele (wettelijke) controle op de onderzoeksresultaten mogelijk blijft. De eerder genoemde lijst met persoonsgegevens en de ID-codes (tot dus ver in bezit van de assistent Dataverzameling) wordt dan onder verantwoordelijkheid van de eindverantwoordelijke onderzoeker, dr. Ernst Bohlmeijer, vernietigd. Vanaf dat moment zijn er alleen anonieme onderzoeksgegevens en daarvoor geldt een bewaarplicht bij het onderzoeksinstituut.

INZAGERECHT. Proefpersonen hebben inzagerecht in de over hen verzamelde gegevens. Zij dienen daartoe een schriftelijk verzoek in te dienen bij de eindverantwoordelijk onderzoeker, dr. Ernst Bohlmeijer. Het verzoek hoeft niet gemotiveerd te worden en zal altijd ingewilligd worden. Elke proefpersoon ontvangt aan het begin van het onderzoek hierover informatie. Van het inzagerecht kan geen gebruik meer worden gemaakt wanneer de persoonsgegevens vernietigd zijn. De onderzoeksgegevens zijn dan anoniem.

CODE GOED GEDRAG. Zowel de onderzoekers als de assistent op de Afdeling Dataverzameling weten zich gebonden door de Code Goed Gedrag, hetgeen een uitwerking is van de privacy wetgeving ter zake.

PROJECTGROEP

Dr. E. Bohlmeijer is universitair hoofddocent bij de Universiteit Twente. In 2007 promoveerde hij op het onderwerp life-review en preventie van depressie bij ouderen. E. Bohlmeijer heeft veel ervaring met het ontwikkelen en onderzoeken van preventieve interventies.

Drs. J. Klungers is preventiefunctionaris bij GGNet. Zij ontwikkelde samen met twee collega's de preventieve mindfulnesstraining.

Drs. W.T.M. Pots is universitair docent bij de Universiteit Twente vanuit een samenwerkingsverband tussen de Universiteit Twente en ADAPT (onderdeel van Dimence). Binnen ADAPT werkt zij als gz-psycholoog en cognitief gedragstherapeut, binnen de ambulante zorg van angst- en stemmingsstoornissen.

REFERENTIES

Baer, R.A. (2003). Mindfulness training as a clinical intervention: A conceptual and empirical review. *Clinical Psychology: Science and Practice, 10*(2), 125-143.

Baer, R.A., Smith, G.T., Hopkins, J., Krietemeyer, J., & Toney, L. (2006). Using self-report assessment methods to explore facets of mindfulness. *Assessment, 13*(1), 27-45.

Beekman, A.T.F., Cuijpers, P., Marwijk, H.W.J. van, Smit, F., Schoevers, R.A & Hosman, C. (2006). Preventie van psychiatrische stoornissen. *Ned Tijdschr Geneeskd, 150*, 419- 423

Bijl, R.V., Ravelli, A., & Van Zessen, G. (1998). Prevalence of psychiatric disorder in the general population: Results of the Netherlands Mental Health Survey and Incidence Study (NEMESIS). *Social Psychiatry and Psychiatric Epidemiology, 33*(12), 587-595.

Bohlmeijer, E. T, Fledderus, M., Pieterse, M.E. (in voorbereiding). The effects of acceptance and commitment therapy as an early intervention on psychological distress in adults: results of a pilot RCT.

Bouma, J., Ranchor, A.V., Sandersman, R. & Van Sonderen, F.L.P. (1995). *Het meten van symptomen van depressie met de CES-D. Een handleiding.* Noordelijk Centrum voor Gezondheidsvraagstukken, Rijksuniversiteit Groningen.

Hayes, S.C. , Luoma, J.B., Bond, F.W., Masuda, A. & Lillis, J. (2006). Acceptance and Commitment Therapy: Model, processes and outcomes. *Behaviour Research and Therapy, 44,* 1-25.

Jacobs, N., Kleen, M., Blokzijl, R., Groot, F. de & A-Tjak, J. (2008). Measuring experiential avoidance. Dutch translation of the Acceptance and Action questionnaire-II (AAQ-II). *Gedragstherapie, 41*, 349-361.

Kabat-Zinn, J. (1990). *Full catastrophe living: Using the wisdom of your body and mind to face stress, pain and illness.* New York: Delacorte.

Keyes, C.L.M. (2005). Mental Illness and/or Mental Health? Investigating Axioms of the Complete State Model of Health. *Journal of Consulting and Clinical Psychology*, *73*(3), 539–548.

Keyes, C.L.M. (2007). Promoting and Protecting Mental Health as Flourishing A Complementary Strategy for Improving National Mental Health. *American Psychologist, 95*(62), 95–108.

Ma, S.H., & Teasdale, J.D. (2004). Mindfulness-Based Cognitive Therapy for Depression: Replication and Exploration of Differential Relapse Prevention Effects. *Journal of Consulting and Clinical Psychology, 72*(1), 31-40.

Meijer, S.A., Smit, F., Schoemaker, C.G. & Cuijpers, P. (2006). *Gezond verstand, evidence-based preventie van psychische stoornissen.* Bilthoven: RIVM.

Moher, D., Jones, A. & Lepage, L. (2001). Use of the CONSORT Statement and Quality of Reports of Randomized Trials. A comparative Before-and-After Evaluation. *Journal of the Americal Midcal Association, 285,* 1992-1995.

Lipsey, M.W. (1990). *Design sensitivity: statistical power for experimental research*. New York: Sage.

Lipsey, M. W., & Wilson, D. B. (1993). The efficacy of psychological, educational, and behavioral treatment: Confirmation from meta-analysis. *American Psychologist, 48,* 1181-1209.

Orsillo, S.M., Roemer, L., & Barlow, D.H. (2003). Integrating Acceptance and Mindfulness into Existing Cognitive-Behavioral Treatment for GAD: A Case Study. *Cognitive and Behavioral Practice, 10*(3), 222-230.

Radloff, L.S. (1977). The CES-D scale: A self-report depression scale for research in the general population. *Applied Psychological Measurement*, 1, 385–401.

Roemer, L., Orsillo, S.M., & Salters-Pedneault, K. (2008). Efficacy of an Acceptance-Based Behavior Therapy for Generalized Anxiety Disorder: Evaluation in a Randomized Controlled Trial. *Journal of Consulting and Clinical Psychology, 76*(6), 1083-1089.

Roemer, L., Lee, J.K., Salters-Pedneault, K., Erisman, S.M., Orsillo, S.M., & Mennin, D.S. (2009). Mindfulness and Emotion Regulation Difficulties in Generalized Anxiety Disorder: Preliminary Evidence for Independent and Overlapping Contributions. *Behavior Therapy, 40*(2), 142-154.

Sheehan. D.V., Janavs, J., Baker R, Harnett-Sheehan, K., Knapp, E., Scheehan, M.F. & Dunbar, G.C. (1998). M.I.N.I. – Mini International Neuropsychiatric Interview- English version 5.0.0. – DSM-IV. *Journal of Clinical psychiatry, 59*, 34-57.

Smit, F., Beekman, A., Cuijpers, P. , De Graaf, R. & Vollebergh, W. (2004). Selecting key variables for depression pervention: results from a population-based prospective epidemiological study. *Journal of Affective Disorders, 81*, 241-249.

Snaith, R.P., (2003) The Hospital Anxiety And Depression Scale. *Health and Quality of Life Outcomes*, 1-29.

Teasdale, J. D., Segal, Z.V., Williams, J.M., Ridgeway, V.A., Soulsby, J.M. & Lau, M.A. (2000). Prevention of relapse/recurrence in major depression by mindfulness-based cognitive therapy. *Journal of Consulting and Clinical Psychology, 68(4),* 615-23.

Westerhof, G.J. & Keyes, C.L.M. (2008). Geestelijke gezondheid is meer dan de afwezigheid van geestelijke ziekte. *Maandblad Geestelijke Gezondheidszorg, 10*(63), 808-820.

Zigmond, A.S. & Snaith, R.P. (1983). The Hospital Anxiety and Depression Scale. Acta *Psychiatrica Scandinavica, 67*, 361–370.

Appendix informatie aandachttraining 'Minder stress door aandacht'

De aandachttraining 'Minder stress door aandacht'

De training bestaat uit 11 bijeenkomsten van anderhalf uur en een follow-up bijeenkomst na 4 of 6 weken. De training uit 11 bijeenkomsten. Centraal staan drie thema's: aandacht, acceptatie en anders omgaan met gedachten. Alle thema's zijn met elkaar verweven, waardoor ze in elke bijeenkomst aan bod komen, ook als ze niet expliciet benoemd worden.

Thema Aandacht
Bijeenkomst 1 Kennismaking
Bijeenkomst 2 Lichaamsscan
Bijeenkomst 3 Ademhaling en informele oefeningen
De eerste drie bijeenkomsten betreffen het thema aandacht. De deelnemers leren hoe ze bewust hun aandacht kunnen richten op het hier en nu. Ook leren ze hoe ze kunnen omgaan met momenten wanneer ze afgeleid raken van datgene waar ze nu mee bezig zijn en weer terug kunnen keren naar dit moment. Er wordt geoefend met twee basisoefeningen: de lichaamsscan en het richten van de aandacht op de ademhaling. We staan uitgebreid stil bij het beoefenen van aandacht in het dagelijks leven. In de eerste bijeenkomst doen we de rozijnoefening (met al je aandacht een rozijn eten). In de derde bijeenkomst bespreken we welke activiteiten en momenten geschikt zijn voor informele oefeningen. In alle volgende weken worden deelnemers gevraagd elke dag een aantal zelfgekozen informele oefeningen te doen. Zowel de ervaringen met het oefenen van de formele als informele oefeningen wordt besproken in elke bijeenkomst van de training.

Thema Omgaan met gedachten
Bijeenkomst 4 Gedachten (1)
Bijeenkomst 6 Gedachten (2)
Bijeenkomst 8 Gedachten (3)
Vanaf bijeenkomst vier wordt om en om een bijeenkomst gewijd aan gedachten en acceptatie. Bij gedachten ligt de nadruk op:
- leren zien dat we niet onze gedachten *zijn*, maar dat we deze gedachten *hebben;*
- leren van een afstand naar deze gedachten te kijken, zodat we ze leren toelaten zonder erin weg te zinken en zonder ze te negeren (observerende zelf);
- ons bewust worden van onze automatische gedachten- en reactiepatronen.

We werken aan het eigen maken van deze vaardigheden met behulp van aandachtsoefeningen en de bespreking van deze oefeningen. Daarnaast wordt de theorie rondom omgaan met gedachten besproken: aandacht voor en acceptatie van gedachten, cognitieve defusie en experiëntele vermijding.

Thema Acceptatie
Bijeenkomst 5 Acceptatie
Bijeenkomst 7 Niet oordelen
Bijeenkomst 9 Vriendelijkheid
Bijeenkomst 10 Loslaten
In deze vier bijeenkomsten leren we dat nú op dit moment alles is zoals het is. De enige mogelijkheid is dit moment toe te laten zoals het is ofwel te accepteren. We staan stil bij de betekenis van de begrippen, zodat helder wordt dat accepteren niet iets is wat 'moet', maar wat vanzelf volgt als we echt aandacht hebben voor wat er nu is. Bij 'Niet oordelen' worden mensen zich bewust hoeveel positieve en negatieve oordelen ze hebben over zichzelf en anderen. Ze leren deze te herkennen, te accepteren en van een afstand te bekijken, zodat ze bewust kunnen kiezen wat ze met deze oordelen doen. Bij 'Vriendelijkheid' oefenen de deelnemers met behulp van een aandachtsoefening vriendelijkheid en mildheid te ervaren voor zichzelf en anderen. Tot slot gaat het bij 'Loslaten' vooral om het leren toelaten van wat er is. De aandachtsoefeningen zijn

afgestemd op deze thema's, de oefeningen worden besproken en de theorie rondom acceptatie wordt besproken.

Bijeenkomst 11 ***Evaluatie***
We kijken terug op wat de training voor een ieder heeft betekend, of deelnemers van plan zijn om verder te gaan met oefenen en zo ja hoe. Daarna wordt besproken wat de meningen van de deelnemers over de training is (inhoud, deelnemersmap, trainers, vorm, praktische zaken). Deze aanbevelingen worden verwerkt in een evaluatieverslag. Waar nodig en mogelijk wordt de training aangepast.

Terugkombijeenkomst *Hoe gaat het nu?*
In deze bijeenkomst bespreken we hoe het iedereen vergaan is na de training. We bespreken het belang van blijven oefenen en oefenen met elkaar. We bekijken met elke deelnemer of hij/zij zelf verder kan of mogelijk behoefte heeft aan aanvullende hulp.
